# Comparison of plasma p‐tau217/Aβ42, p‐tau217, and Aβ42/Aβ40 biomarkers by race to detect Alzheimer's disease

**DOI:** 10.1002/alz.70469

**Published:** 2025-08-13

**Authors:** Katheryn A. Q. Cousins, Magdalena Korecka, Yang Wan, Amberley Vulaj, Christopher Brown, Thomas F. Tropea, Edward B. Lee, Duygu Tosun, Susan M. Landau, Ozioma C. Okonkwo, Monica Rivera Mindt, Michael W. Weiner, David J. Irwin, David A. Wolk, Leslie M. Shaw, and the Alzheimer's Disease Neuroimaging Initiative

**Affiliations:** ^1^ Department of Neurology Perelman School of Medicine University of Pennsylvania Philadelphia Pennsylvania USA; ^2^ Department of Pathology and Laboratory Medicine Perelman School of Medicine University of Pennsylvania Philadelphia Pennsylvania USA; ^3^ Department of Radiology and Biomedical Imaging University of California San Francisco California USA; ^4^ Neuroscience Department University of California Berkeley California USA; ^5^ Department of Medicine and the Wisconsin Alzheimer's Disease Research Center University of Wisconsin Madison Wisconsin USA; ^6^ Department of Psychology Fordham University New York New York USA; ^7^ Department of Neurology Icahn School of Medicine at Mount Sinai New York New York USA; ^8^ Northern California Institute for Research & Education San Francisco California USA; ^9^ Departments of Radiology Medicine, Psychiatry & Neurology University of California San Francisco San Francisco California USA; ^10^ Center for Imaging of Neurodegenerative Diseases San Francisco Veterans Administration Medical Center San Francisco California USA

**Keywords:** Alzheimer's disease, Aβ42/Aβ40, cognition, differential diagnosis, plasma biomarkers, p‐tau217, p‐tau217/Aβ42, race, test–retest

## Abstract

**INTRODUCTION:**

In clinically and racially diverse training and test sets, we evaluated and validated Alzheimer's disease (AD) plasma biomarkers: phosphorylated tau‐217 (p‐tau217), amyloid beta 1‐42/1‐40 (Aβ42/Aβ40), and p‐tau217/Aβ42.

**METHODS:**

Inclusion criteria were available plasma, race (people who self‐identified as Black/African American [pBlack] or White [pWhite]), cognitive status (normal, mild cognitive impairment [MCI], dementia), and ^18^F‐florbetaben or ^18^F‐florbetapir positron emission tomography (PET) data. In the training set (*n* = 289; 28% pBlack), we used receiver‐operating characteristic (ROC) analysis to calculate two cut points (0.95 sensitivity and specificity) to detect amyloid PET positivity. Cut points were validated in an independent test set (*n* = 846; 12% pBlack).

**RESULTS:**

In the test set, plasma p‐tau217/Aβ42 had the highest accuracy (pBlack: 0.88, pWhite: 0.91) and lowest proportion of intermediate classifications (≤0.16), with no classification difference by race (odds ratio [OR] = 1.45, 95% confidence interval [CI] = 0.66–2.95, *p *= 0.33). False negatives were more likely to be cognitively normal (vs mild cognitive impairment [MCI]; OR = 7.79, 95% CI = 2.33–40.81, *p *= 5.1e‐05) than correct classifications.

**DISCUSSION:**

Plasma p‐tau217/Aβ42 had high accuracy to detect AD, with comparable performance across race.

**Highlights:**

- We tested plasma phosphorylated tau‐217 (p‐tau217)/amyloid beta (Aβ)_42_, p‐tau217, and Aβ42/Aβ40 in diverse training and test sets.
- In models, race × amyloid PET interactions were not significant.
- Plasma p‐tau217/Aβ42 had the highest accuracy in people who self‐identified as Black/African American (pBlack; 0.88) and people who self‐identified as White (pWhite; 0.91).
- Plasma p‐tau217/Aβ42 had the lowest proportion of intermediate cases (range = 0.09–0.16).
- False negatives were more likely to have normal cognition, female sex, and high body mass index.

## 1 BACKGROUND

Plasma biomarkers are promising tools to detect Alzheimer's disease (AD) pathology,^1^, ^2^, ^3^, ^4^ with plasma phosphorylated tau‐217 (p‐tau217) being recently recommended as a core AD biomarker.^5^ In head‐to‐head comparisons, plasma biomarkers measured by Fujirebio Lumipulse had excellent performance, comparable to mass spectrometry^6^; combining plasma p‐tau217 with amyloid beta 1‐42/1‐40 (Aβ42/Aβ40) further improved AD diagnostic performance.^6^, ^7^, ^8^ These studies and others rigorously compare blood biomarkers to gold standard amyloid positron emission tomography (PET) or autopsy data.^9^, ^10^ However, such comparisons with gold standard data are largely in demographically homogeneous samples, although with some progress.^11^, ^12^, ^13^, ^14^ Before clinical implementation, continued blood biomarker validation in diverse samples is needed^15^, ^16^ to ensure generalizability of biomarkers and accurate interpretation in the larger population.

One understudied dimension is racial diversity, where findings are mixed. Although we do not have an a priori hypothesis that genetic ancestry affects biomarker levels,^17^ some factors that affect biomarkers and AD risk (e.g., comorbidities, social/structural determinants of health [SSDOH]) may associate with racial identity.^15^ Some find higher plasma Aβ42/Aβ40 in people who self‐identify as Black/African American (pBlack) compared to White (pWhite),^18^, ^19^ or find differences in other plasma biomarkers or ethnicities.^14^, ^18^, ^20^ Others find no differences in plasma Aβ42/Aβ40^20^, ^21^ or p‐tau217 levels^12^, ^14^ by race after controlling for potential confounds. To date, no studies establish biomarker cut points in a diverse sample and then validate in an independent sample to directly test classification accuracy across race.

Another dimension to biomarker performance is the prevalence of AD pathology in the tested population, since the positive predictive value (PPV) of a biomarker decreases as incidence decreases.^22^, ^23^ Expanded testing using plasma biomarkers would include populations with lower AD incidence or mixed clinical status, which will influence interpretation.^24^ Likewise, cognitive status and amyloid prevalence can differ by race.^25^, ^26^ Thus it is important to test biomarkers across clinical phases that include unimpaired/normal cognition,^7^, ^27^ mild cognitive impairment (MCI), ^4^ and dementia.^28^

Here we investigate how race (pBlack, pWhite) and cognitive status (normal, MCI, dementia) affect biomarker performance. Fujirebio Lumipulse G1200 measured concentrations of p‐tau217, Aβ42, and Aβ40 in plasma; amyloid PET determined amyloid status (±).^5^, ^29^ Part 1 tested AD plasma p‐tau217, p‐tau217/Aβ42, and Aβ42/Aβ40 in a training set from the University of Pennsylvania (UPenn). To increase classification confidence, we followed recent recommendations to implement a two‐threshold approach.^23^ Thus, receiver‐operating characteristic (ROC) analyses determined ≥0.95 sensitivity and ≥0.95 specificity cut points. These two thresholds result in three classifications: Aβ positive (Aβ+), Aβ negative (Aβ–), and intermediate Aβ status for values between these thresholds. In Part 2, linear models tested plasma biomarker differences by race and interactions with amyloid severity; to account for potential sampling bias, propensity scores rigorously matched pBlack and pWhite for amyloid PET severity and demographic factors. In Part 3, we validated cut points in an independent test set from the Alzheimer's Disease Neuroimaging Initiative (ADNI) study and compared performance by race.

## 2 METHODS

For all participants, selection criteria were based on available banked plasma samples, clinical diagnosis, amyloid PET (^18^F‐florbetaben [UPenn and ADNI] or ^18^F‐florbetapir [ADNI only]), and self‐reported race.

**Training set**. Participants (*n* = 289; 28% pBlack) were recruited for observational research at the UPenn ADRC and selected from the Integrative Neurodegenerative Disease Database.^30^, ^31^ Written informed consent was obtained in accordance with the Declaration of Helsinki and UPenn Institutional Review Board.

**Test set**. Participants were selected from ADNI (*n* = 846; 12% pBlack). Data used in the preparation of this article were obtained from the ADNI database (adni.loni.usc.edu). ADNI was launched in 2004 as a public–private partnership, led by Principal Investigator Michael W. Weiner, MD. The primary goal of ADNI has been to test whether serial magnetic resonance imaging (MRI), PET, other biological markers, and clinical and neuropsychological assessment can be combined to measure the progression of MCI and early AD.

### 2.1 Plasma collection and analysis

Whole blood samples were collected in ethylenediaminetetraacetic acid (EDTA) tubes and spun down at 3000 rpm for 15 min at 4°C; resulting plasma fractions were aliquoted into polypropylene tubes, placed on dry ice, and stored at −80°C within 24 hours of collection according to standard procedures^31^ (see documentation https://adni.loni.usc.edu). On the Fujirebio Lumipulse G1200 automated immunoassay platform, plasma samples were analyzed in singlicate and quantified using Fujirebio chemiluminescence immunoassay reagents for Aβ42, Aβ40, and p‐tau217. Reagents exist within separate compartments in individual test immunoreaction cartridges (see package inserts for Lumipulse G plasma Fujirebio Europe NV). For the p‐tau217 assay, the automated procedure includes introduction of capture anti‐p‐tau217 monoclonal antibody‐linked to ferrite beads, and binding to the antibody of p‐tau217 in the plasma sample, calibration standard, or quality control sample. Following a wash/rinse cycle of the p‐tau217 antibody beads complexed with p‐tau217, the second step adds alkaline phosphatase–labeled anti‐tau monoclonal antibodies, which bind to the bead‐linked immunocomplexes. Following a wash/rinse cycle, substrate solution containing the following is mixed with the bead complexes: biotinylated detector monoclonal antibody, an enzyme‐linked Streptavidin solution, and the enzyme substrate 3‐(2′‐spiroadamantane)‐4‐methoxy‐4‐(3″‐phosphoryloxy)phenyl‐1,2‐dioxetane disodium salt (AMPPD). The luminescence signal generated by de‐phosphorylated AMPPD, detectable at 477 nm wavelength, reflects the amount of p‐tau217. Immunoassays for Aβ42 and Aβ40 follow the same scheme except for analyte‐specific antibodies.

**RESEARCH IN CONTEXT**

1. **Systematic review**: Literature reviews (e.g., PubMed, Google scholar) show varied findings in whether Alzheimer's disease (AD) plasma biomarkers (phosphorylated tau‐217 [p‐tau217]/amyloid beta [Aβ]_42_, p‐tau217, Aβ42/Aβ40) differ by self‐reported race. To date, no studies establish biomarker cut points in a diverse sample and then validate in an independent sample to directly test classification accuracy across race.
2. **Interpretation**: Propensity scores matched people who self‐identify as White (pWhite) to people who self‐identify as Black/African American (pBlack) by amyloid positron emission tomography (PET) severity and demographic factors, and linear models showed that the relationship between biomarkers and amyloid severity (PET) did not differ by race (non‐significant race × amyloid PET interactions). In the test set, we find that plasma p‐tau217/Aβ42 had the highest accuracy in pBlack and pWhite and lowest proportion intermediate, compared to p‐tau217 and Aβ42/Aβ40. False negatives by p‐tau217/Aβ42 were more likely to be cognitively normal (vs mild cognitive impairment), female, and with a higher body mass index than correct classifications.
3. **Future directions**: Accuracy of plasma p‐tau217/Aβ42 should be further assessed in real‐world datasets.

We documented analytical performance, including run‐to‐run precision and lot‐to‐lot performance for p‐tau217, since two different reagent lots were used for the ADNI4 “in‐clinic” + UPenn #1 batch plasma samples and the ADNI “legacy” + UPenn #2 batch samples. The linear regression—*Y(lot #D4C4129/1) = 0.9962∗X(lot#D4C4059)–0.003102*—and *r* ^2 ^= 0.989 indicate excellent lot‐to‐lot performance for the 84 plasma samples used for this analysis. The same reagent lots were used for Aβ42 and Aβ40 for all study samples. Precision across the multiple runs for the two plasma pools: Aβ42 range = 5.1%–5.4%; Aβ40 = 3.7%–4.2%; and p‐tau217 = 6.2%–9.9%. Test/re‐test performance is summarized in eTable 1.

### 2.2 Amyloid status and severity

In the UPenn dataset, amyloid status (±) was determined by visual reads of the closest available ^18^F‐florbetaben amyloid PET, performed by expert readers, according to previous methods.^32^ We computed mean amyloid standardized uptake value ratio (SUVR) within the neocortex, defined by the ANTs 6‐class tissue segmentation^33^ and divided by mean signal in a whole‐cerebellum reference region. Time between plasma collection and PET (plasma‐to‐PET interval) averaged 0.5 years (standard deviation [SD] = 0.5).

For ADNI, amyloid status was determined by Centiloids ≥25,^34^, ^35^ and severity was computed using Centiloids. Plasma‐to‐PET interval averaged 0.7 years (SD = 1.6).

### 2.3 Demographic, clinical, and neuropsychological assessments

Race, ethnicity, age, disease onset (first reported symptom), and sex were collected by self‐report. Area Deprivation Index (ADI)—a neighborhood‐level composite of education, employment, housing‐quality, and poverty—was determined using the Neighborhood Atlas, based on national data (2021 Area Deprivation Index v4.01: https://www.neighborhoodatlas.medicine.wisc.edu/),^36^ and tertiled (high = 66–100, medium = 34–65, low = 0–33). Body mass index (BMI) was calculated from weight (pounds) and height (inches): *weight × 708/height^2^*. Apolipoprotein E (*APOE*) genotyping was used.^37^ For the UPenn sample (not available for ADNI), histories of diabetes, heart attack, stroke, and traumatic brain injury were determined using self‐report or clinician report; one person (amyloid+ MCI) had a reported history of chronic kidney disease, stage IV (p‐tau217/Aβ42 = 0.078, p‐tau217 = 3.628, Aβ42/Aβ40 = 0.079). For ADNI (not available for UPenn), blood creatinine mg/dL measured kidney dysfunction.^13^, ^38^, ^39^

Racial identity categories were White (209 UPenn; 741 ADNI) and Black/African American (80 UPenn; 105 ADNI [including 1 American Indian/Black, and 1 Black/White/Hispanic]).

Clinical syndrome was determined according to National Alzheimer's Coordinating Center (NACC) Uniform Dataset 3.0 criteria^40^ at the time of plasma collection. Participants were cognitively normal (178 UPenn [including six impaired without MCI]; 463 ADNI), MCI (80 UPenn; 383 ADNI), or dementia (31 UPenn; 0 ADNI), which included amnestic multidomain dementia, posterior cortical atrophy, primary progressive aphasia, frontotemporal dementia, Lewy body spectrum diseases, and non‐amnestic multidomain dementia.

### 2.4 Statistical analyses

Variables were not normally distributed; non‐parametric tests (e.g., Mann–Whitney–Wilcoxon, Spearman's rho) were used in unadjusted comparisons, reported in Figures 2 and 3. In linear models, plasma biomarkers were log‐transformed, and β‐estimates, 95% confidence intervals (95% CIs), and *p*‐values were reported. The significance threshold was α = 0.05. In addition, Bonferroni correction for multiple comparisons across three biomarkers (α = 0.0167) was also reported. Analyses were conducted using R version 4.4.2 (2024‐10‐31) software.

**Missing data**. To maximize sample size, variables with any missing data in pBlack (e.g., BMI, ADI, *APOE* ε4, and creatinine; see Table 1) were not included in propensity score matching (below). Missing data were dropped listwise from models; thus variables with a high percentage of missing data in pBlack (≥20%)—BMI in UPenn (*n* = 16 [20%]) and ADI in ADNI (*n* = 43 [41%])—were not included as covariates.

**TABLE 1:** Demographic and clinical characteristics of training set and matched participants. For continuous variables, median and interquartile range (IQR) are reported; Kruskal–Wallis tests performed group comparisons. For categorical variables, count (percentage [%]) are provided; chi‐square tests performed frequency comparisons. *p*‐values are reported for group comparisons. (A) UPenn participants; training set (*n* = 289). (B) Subset of UPenn participants selected using propensity score matching (*n* = 160).

A. UPenn training set	White	Black	*p*	Missing
*n*	209	80		
PET status = amyloid+ (%)	99 (47.4%)	21 (26.2%)	0.002	–
Aβ PET SUVR	1.1 [0.9, 1.4]	1.0 [0.9, 1.1]	0.006	–
Cognitive status (%)			< 0.001	–
Normal	113 (54.1%)	65 (81.2%)		
MCI	71 (34.0%)	9 (11.2%)		
Dementia	25 (12.0%)	6 (7.5%)		
Age at plasma (years)	73.0 [69.0, 78.0]	72.0 [69.0, 76.0]	0.547	–
Body mass index (BMI)	25.7 [22.7, 29.0]	29.7 [26.2, 34.6]	< 0.001	16
Sex = male (%)	101 (48.3%)	9 (11.2%)	< 0.001	–
Ethnicity = Hispanic or Latino (%)	2 (1.0%)	0	0.932	–
Education	18.0 [16.0, 18.0]	16.0 [12.8, 18.0]	< 0.001	–
*APOE* = ≥1 ε4 allele(s) (%)	82 (39.2%)	34 (43.0%)	0.651	1
Area Deprivation Index (ADI) (%)			< 0.001	21
Low deprivation	127 (66.5%)	20 (26.0%)		
Medium deprivation	58 (30.4%)	29 (37.7%)		
High deprivation	6 (3.1%)	28 (36.4%)		
History of diabetes (%)	25 (12.0%)	25 (31.2%)	< 0.001	–
History of heart attack (%)	11 (5.3%)	1 (1.2%)	0.230	–
History of stroke (%)	2 (1.0%)	1 (1.2%)	1.000	2
History of traumatic brain injury (%)	28 (16.9%)	7 (11.1%)	0.381	60

B. UPenn matched cases	White	Black	*p*	Missing
*n*	80	80		
PET status = amyloid+ (%)	24 (30.0%)	21 (26.2%)	0.725	–
Aβ PET SUVR	1.0 [0.9, 1.2]	1.0 [0.9, 1.1]	0.692	–
Cognitive status (%)			0.853	–
Normal	63 (78.8%)	65 (81.2%)		
MCI	9 (11.2%)	9 (11.2%)		
Dementia	8 (10.0%)	6 (7.5%)		
Age at plasma (years)	72.0 [69.0, 76.0]	72.0 [69.0, 76.0]	0.899	–
Body mass index (BMI)	24.7 [22.2, 29.8]	29.7 [26.2, 34.6]	< 0.001	16
Sex = male (%)	10 (12.5%)	9 (11.2%)	1.000	–
Ethnicity = not Hispanic or Latino (%)	80 (100.0%)	80 (100.0%)	–	–
Education	16.0 [14.0, 18.0]	16.0 [12.8, 18.0]	0.068	–
*APOE* = ≥1 ε4 allele(s) (%)	22 (27.5%)	34 (43.0%)	0.059	1
Area Deprivation Index (ADI) (%)			< 0.001	10
Low deprivation	47 (64.4%)	20 (26.0%)		
Medium deprivation	23 (31.5%)	29 (37.7%)		
High deprivation	3 (4.1%)	28 (36.4%)		
History of diabetes (%)	13 (16.2%)	25 (31.2%)	0.041	–
History of heart attack (%)	1 (1.2%)	1 (1.2%)	1.000	–
History of stroke (%)	1 (1.2%)	1 (1.2%)	1.000	–
History of traumatic brain injury (%)	5 (8.3%)	7 (11.1%)	0.830	37

***Outliers***. Two UPenn amyloid‐negative cases with normal cognition had plasma p‐tau217 levels above 1.0 pg/mL (amyloid negative p‐tau217 median = 0.087; interquartile range [IQR] = 0.058). Both cases had repeated measurements with similarly elevated p‐tau217: (1) 1.73 pg/mL versus 1.30 pg/mL collected 3.7 years later, with progression to MCI; and (2) 3.76 pg/mL versus 4.89 pg/mL 3.0 years later, with no clinical progression observed after 4.1 years. No irregularities were observed, and these cases were included in analyses.

#### 2.4.1 Part 1: Training set (all UPenn)

Part 1 evaluates plasma biomarkers in all UPenn samples. ROC and area under the curve (AUC) with bootstrapping (2000 iterations) tested biomarker accuracy.^41^ Thresholds were determined at ≥95% sensitivity and ≥95% specificity; thresholds were not stratified by race. ROC analyses were repeated within cognitively normal and MCI/dementia.^22^, ^23^

Linear models compared biomarkers (dependent variable) by amyloid status (±), race (pWhite, pBlack), and cognitive status (normal, MCI, dementia); models covaried for factors that might affect plasma concentrations (age, sex, *APOE* ε4 [0 vs ≥1]) or that differed by race (ADI, history of diabetes; see Table 1). Effect sizes were calculated using generalized η^2^ (η^2^ *_G_*) and partial sum of squares^42^ (≥0.14 large; ≥0.06 medium; ≥0.01 small).^43^

#### 2.4.2 Part 2: Matched UPenn set

To ensure that observed differences by race were not due to differences in amyloid severity or other factors, propensity score matching selected pWhite and pBlack matched for amyloid PET SUVR, cognitive status, age, sex, education, and history of diabetes. Linear models were repeated in matched cases, and an interaction term (amyloid PET SUVR × race) tested race‐specific differences in the association between plasma biomarkers and amyloid pathology.

#### 2.4.3 Part 3: Test set (ADNI)

We validated accuracy of UPenn‐derived cut points in both UPenn and ADNI datasets. Performance metrics were accuracy, sensitivity, specificity, PPV, and negative predictive value (NPV); substandard performance was considered <0.80.

As above, propensity scores matched ADNI pWhite to pBlack for amyloid severity (PET Centiloids), cognitive status, age, education, and sex. Fisher's exact tests (odds ratio [OR]) investigated differences in plasma classification accuracy (correct vs errors) between pBlack and pWhite in matched ADNI. Linear models compared biomarker levels by race, covarying for amyloid status, age, sex, *APOE* ε4, BMI, and creatinine.

## 3 RESULTS

Table 1 summarizes the clinical and demographic characteristics of participants stratified by race, for the full UPenn (Table 1) and matched samples (Table 1).

### 3.1 Part 1: Training set (UPenn)

**FIGURE 1:**
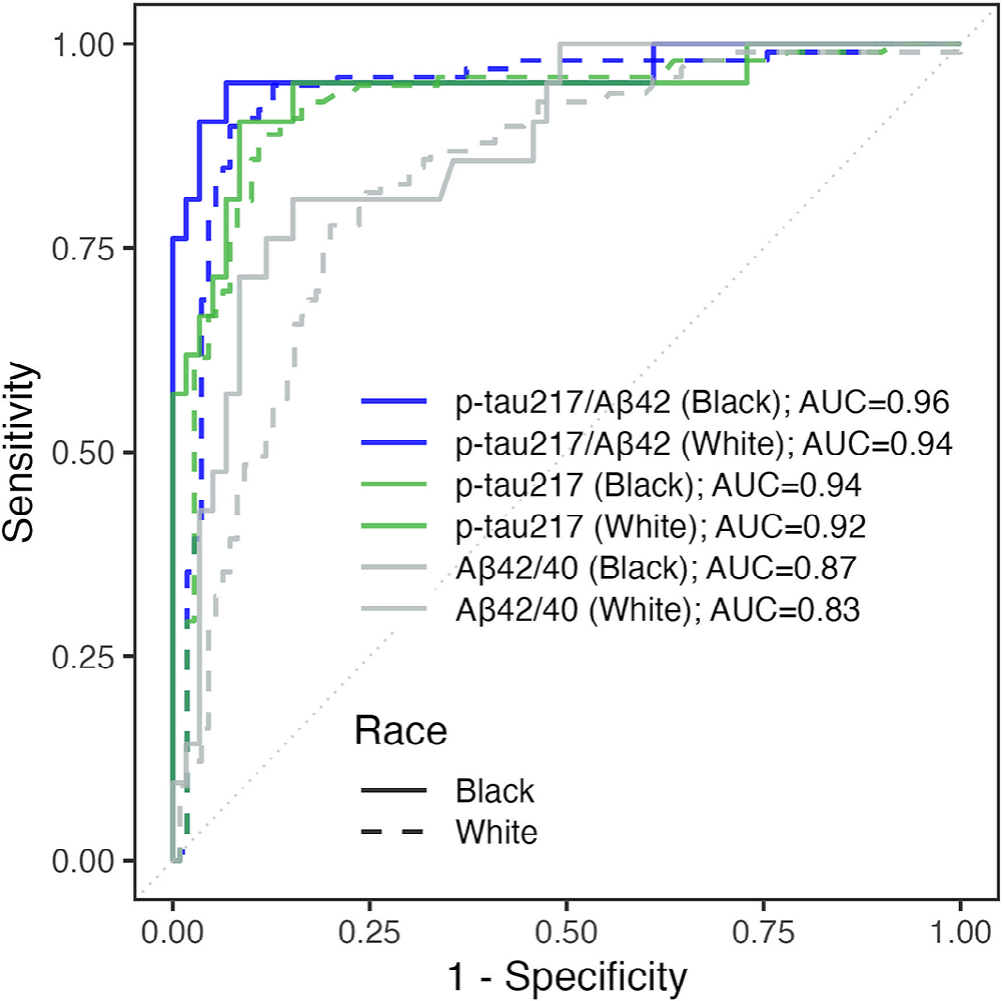
Receiver‐operating characteristic (ROC) curves to discriminate amyloid positive from amyloid negative in the training set (*n* = 289). ROC curves plot sensitivity versus specificity. Color indicates biomarker: p‐tau217/Aβ42 (blue), p‐tau217 (green), and Aβ42/Aβ40 (gray). Linetype indicates race: Black (solid; *n* = 80), White (broken; *n* = 209). p‐tau217, phosphorylated tau at 217; Aβ, β‐amyloid.

ROC analyses compared classification accuracy for plasma biomarkers and determined 95% sensitivity and 95% specificity thresholds (Table 2), showing the highest overall performance for plasma p‐tau217/Aβ42 (AUC = 0.95; Table 2), consistent in both pBlack (AUC = 0.96; Figure 1) and pWhite (AUC = 0.94; Figure 1). ROC analyses also tested biomarkers and derived cut points within cognitively normal (Table 2) and MCI/dementia (Table 2); AUCs were higher in MCI/dementia than in normal (Figures S1 and S2).

**TABLE 2:** Receiver‐operating characteristic (ROC) analyses to discriminate PET amyloid‐positive from amyloid negative in training set (All; *n* = 289), normal cognition (*n* = 178), and MCI/dementia (*n* = 111).

A. UPenn training set (all)	AUC	AUC 95% CI	Method	Threshold	Sensitivity	Specificity	Accuracy
Plasma p‐tau217/Aβ42	0.95	0.91–0.97	≥0.95 sens	0.0055	0.95	0.87	0.90
			≥0.95 spec	0.0086	0.84	0.95	0.91
Plasma p‐tau217 (pg/mL)	0.93	0.89–0.96	≥0.95 sens	0.1280	0.95	0.77	0.85
			≥0.95 spec	0.3000	0.67	0.95	0.83
Plasma Aβ42/Aβ40	0.85	0.80–0.89	≥0.95 sens	0.1053	0.95	0.44	0.65
			≥0.95 spec	0.0820	0.34	0.95	0.70

B. UPenn: normal cognition	AUC	AUC 95% CI	Method	Threshold	Sensitivity	Specificity	Accuracy
Plasma p‐tau217/Aβ42	0.89	0.81–0.94	≥0.95 sens	0.0022	0.95	0.33	0.47
			≥0.95 spec	0.0087	0.61	0.96	0.88
Plasma Aβ42/Aβ40	0.85	0.78–0.91	≥0.95 sens	0.1026	0.95	0.53	0.63
			≥0.95 spec	0.0776	0.17	0.96	0.77
Plasma p‐tau217 (pg/mL)	0.85	0.77–0.91	≥0.95 sens	0.0660	0.95	0.27	0.43
			≥0.95 spec	0.2890	0.39	0.96	0.83

C. UPenn: MCI/dementia	AUC	AUC 95% CI	Method	Threshold	Sensitivity	Specificity	Accuracy
Plasma p‐tau217/Aβ42	0.98	0.94–1.00	≥0.95 sens	0.0086	0.96	0.94	0.96
			≥0.95 spec	0.0108	0.84	0.97	0.87
Plasma p‐tau217 (pg/mL)	0.97	0.94–0.99	≥0.95 sens	0.1970	0.96	0.88	0.94
			≥0.95 spec	0.3340	0.77	0.97	0.83
Plasma Aβ42/Aβ40	0.85	0.77–0.92	≥0.95 sens	0.1069	0.96	0.31	0.77
			≥0.95 spec	0.0861	0.49	0.97	0.63

Bootstrapping with 2000 iterations computed ROC metrics across all training set participants. Area under the curve (AUC) and 95% confidence interval (95% CI) for each biomarker are reported. Thresholds are determined at two levels: Value at 0.95 sensitivity (Method: 0.95 sens) and 0.95 specificity (Method: 0.95 spec); sensitivity, specificity, and accuracy at each threshold are reported. We note that thresholds were derived for the full training sample; race‐specific thresholds were not computed.

Abbreviations: Aβ, β‐amyloid; MCI, mild cognitive impairment; p‐tau217, phosphorylated tau at 217; UPenn, University of Pennsylvania.

**FIGURE 2:**
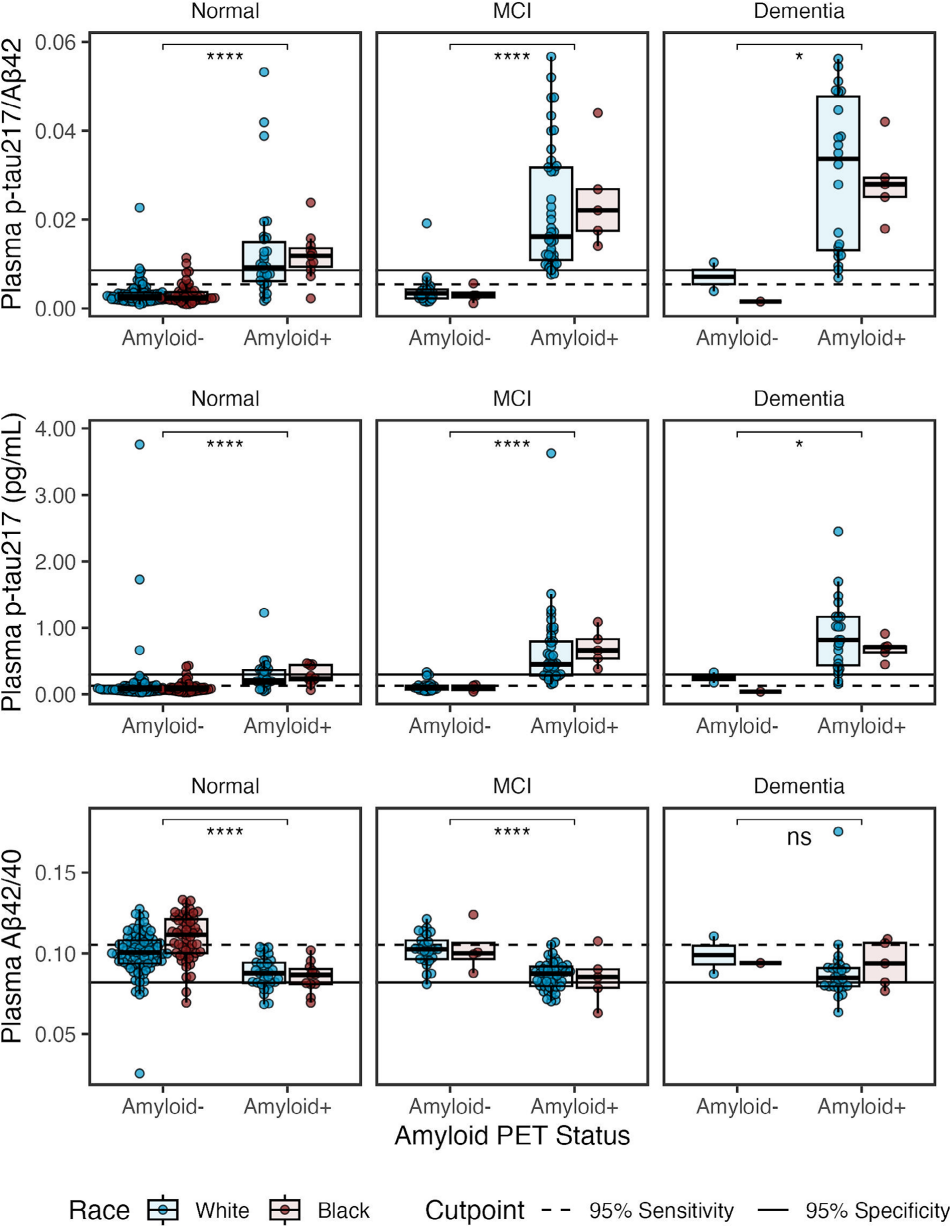
Plasma biomarkers by race (White, Black), amyloid status (amyloid positive, amyloid negative), and cognitive status (normal, MCI, dementia) in all UPenn samples (*n* = 289). Boxplots show median, interquartile range (IQR), and outliers for each plasma biomarker. Color indicates pWhite (blue; *n* = 209) and pBlack (red; *n* = 80). Panels show cognitively normal (left; *n* = 178), MCI (middle; *n* = 80), and dementia (right; *n* = 31); right panels are individuals with cognitive impairment. Solid horizontal line indicates 0.95 specificity threshold; broken horizonal lines indicate 0.95 sensitivity threshold (Table 2). Asterisks represent Bonferroni adjusted *p*‐values from Wilcoxon pairwise comparisons (*p < 0.05; **** *p *< 0.0001).

We next examined group differences in biomarker levels (Figure 2); mean and SDs for all biomarkers are listed in Table S2. Linear models with covariates (Table S3) showed that amyloid positivity was associated with higher plasma p‐tau217/Aβ42 (β = 1.4, 95% CI = 1.2–1.6, *p *= 1.5e‐30; Bonferroni‐*p *= 4.4e‐30) with large effect (η^2^ *_G _* = 0.40), higher p‐tau217 (β = 1.2, 95% CI = 0.99–1.4, *p *= 1.7e‐24; Bonferroni‐*p *= 5.1e‐24) with large effect (η^2^ *_G _* = 0.34), and lower Aβ42/Aβ40 (β = −0.14, 95% CI = −0.18 to −0.09, *p *= 1.3e‐08; Bonferroni‐*p *= 4e‐08) with medium effect (η^2^ *_G _* = 0.12). Cognitive status was associated with higher plasma p‐tau217/Aβ42 with medium effect (η^2^ *_G _* = 0.087) (MCI: β = 0.31, 95% CI = 0.1–0.52, *p *= 0.0036, Bonferroni‐*p *= 0.011; dementia: β = 0.74, 95% CI = 0.43–1, *p *= 2.9e‐06, Bonferroni‐*p *= 8.7e‐06), and higher p‐tau217 with medium effect (η^2^ *_G _* = 0.12) (MCI: β = 0.37, 95% CI = 0.16–0.58, *p *= 0.00053, Bonferroni‐*p *= 0.0016; dementia: β = 0.86, 95% CI = 0.56–1.2, *p *= 4.1e‐08, Bonferroni‐*p *= 1.2e‐07); Aβ42/Aβ40 did not differ by cognitive status (MCI: *p *= 0.53; dementia: *p *= 0.34). Although neither p‐tau217/Aβ42 (*p *= 0.67) nor p‐tau217 (*p *= 0.28) differed by race, pBlack had higher plasma Aβ42/Aβ40 levels than pWhite (β = 0.085, 95% CI = 0.037–0.13, *p *= 0.00063; Bonferroni‐*p *= 0.0019) with small effect (η^2^ *_G _* = 0.045).

Neither plasma Aβ42 (β = 0.072, 95% CI = −0.0025 to 0.15, *p *= 0.058) nor Aβ40 (*p *= 0.66) differed by race (Figure S3; Table S3).

Because BMI may affect plasma levels,^38^ supplementary analyses re‐tested models including BMI as a covariate; BMI was not significant and did not affect main results (Table S3).

### 3.2 Part 2: Matched UPenn set

**FIGURE 3:**
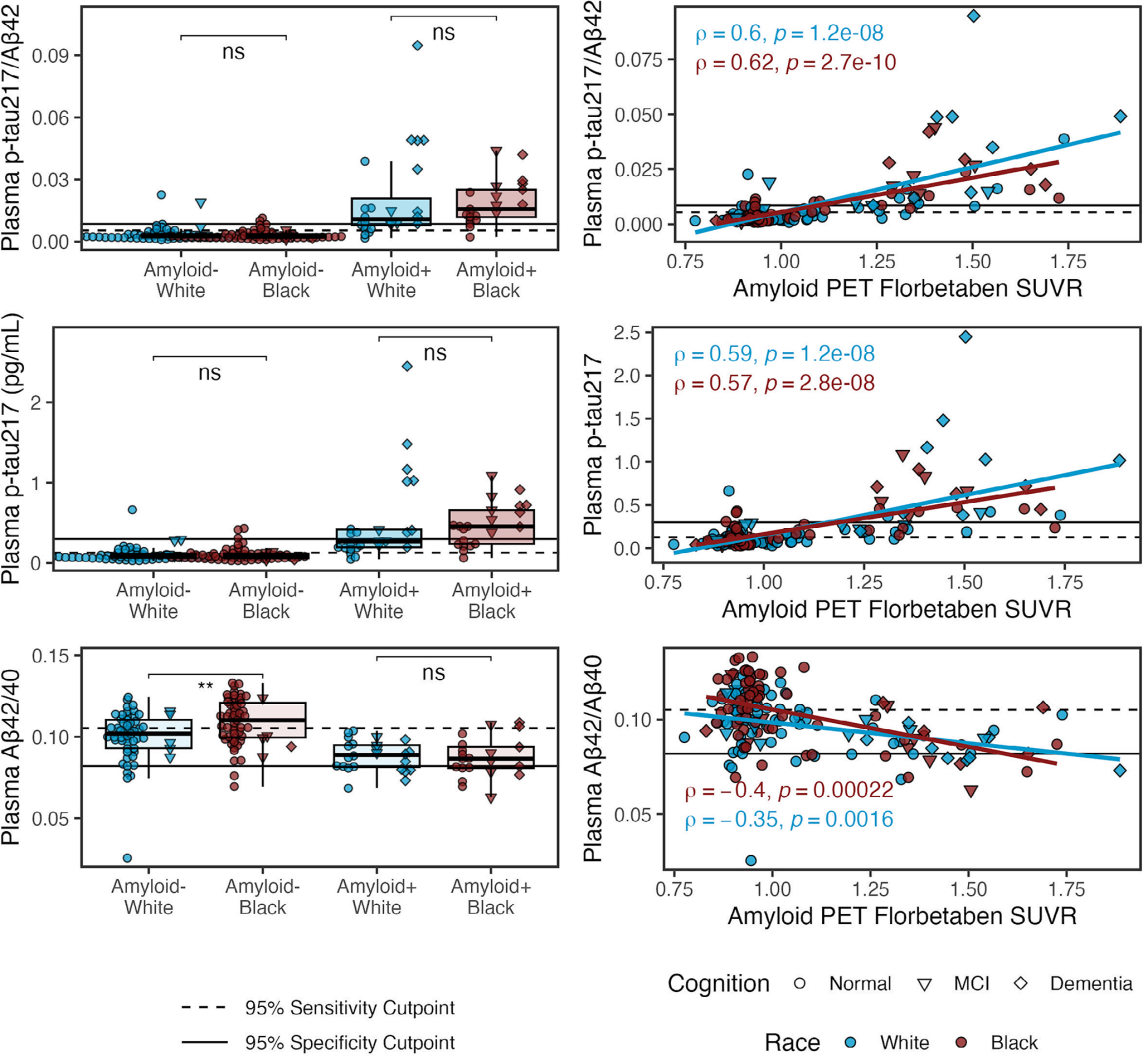
Plasma biomarkers by race (White, Black) and amyloid status (±) in matched sample (*n* = 160). (Left panels.) Boxplots show median, interquartile range (IQR), and outliers for each plasma biomarker. Color indicates pWhite (blue; *n* = 209) and pBlack (red; *n* = 80); shape indicates cognitive status of normal (circle; *n* = 178), MCI (triangle; *n* = 80), or dementia (diamond; *n* = 31). Solid horizontal line indicates 0.95 specificity threshold; broken horizonal lines indicate 0.95 sensitivity threshold (Table 1). Asterisks represent Bonferroni adjusted *p*‐values from Wilcoxon pairwise comparisons (*** Bonferroni‐*p *< 0.001 or not significant [ns]). (Right panels.) Scatterplot of plasma biomarkers association with amyloid PET SUVR. Least squares regression lines are plotted. Spearman's correlations are reported with nominal *p*‐values. Excluded one p‐tau217 outlier (3.76 pg/mL in White amyloid‐negative individual) from figures for improved visualization, but was not excluded from statistical analyses.

In pBlack and pWhite who were amyloid and demographically matched (Table 1, Figure 3), linear models with covariates (Table S4) confirmed that pBlack had higher plasma Aβ42/Aβ40 levels than pWhite (β = 0.11, 95% CI = 0.047–0.17, *p *= 0.00059; Bonferroni‐*p *= 0.0018) with medium effect (η^2^ *_G _* = 0.082), with the largest differences in the amyloid‐negative group (see Figure 3). Neither p‐tau217 (*p *= 0.37) nor p‐tau217/Aβ42 (*p *= 0.95) differed by race.

However, interactions (race × amyloid PET SUVR)—which tested if the relationship between amyloid burden (PET SUVR) and plasma levels differed by race—were not significant (all *p*’s ≥ 0.21; Table S5).

### 3.3 Part 3: Test set (ADNI) validation

Table 3 summarizes the clinical and demographic characteristics of participants stratified by race for ADNI samples.

**TABLE 3 alz70469-tbl-0003:** Demographic and clinical characteristics of ADNI test set participants (*n* = 846).

ADNI Test Set	White	Black	p	Missing
*n*	741	105		
Pet Status = amyloid+ (%)	347 (46.8%)	36 (34.3%)	0.021	–
Aβ PET Centiloid	17.0 [1.0, 81.0]	9.0 [−1.0, 41.0]	0.005	–
Cognitive status = MCI (%)	348 (47.0%)	35 (33.3%)	0.012	–
Age at plasma (years)	74.0 [69.0, 79.0]	70.0 [65.0, 75.0]	<0.001	–
Education	16.0 [15.0, 18.0]	16.0 [14.0, 18.0]	0.003	–
Body mass index (BMI)	26.4 [23.7, 29.6]	29.5 [25.5, 33.9]	<0.001	127
Creatinine	0.9 [0.8, 1.1]	0.9 [0.7, 1.0]	0.175	210
Sex = male (%)	397 (53.6%)	28 (26.7%)	<0.001	–
*APOE* = ≥1 ε4 allele(s) (%)	312 (43.5%)	40 (42.1%)	0.881	34
Area Deprivation Index (ADI) (%)			0.003	519
Low deprivation	148 (55.8%)	24 (38.7%)		
Medium deprivation	79 (29.8%)	18 (29.0%)		
High deprivation	38 (14.3%)	20 (32.3%)		

For continuous variables, median and interquartile range (IQR) are reported; Kruskal–Wallis tests performed group comparisons. For categorical variables, counts (percentage [%]) are provided; chi‐square tests performed frequency comparisons. *p*‐values are reported for group comparisons.

**FIGURE 4 alz70469-fig-0004:**
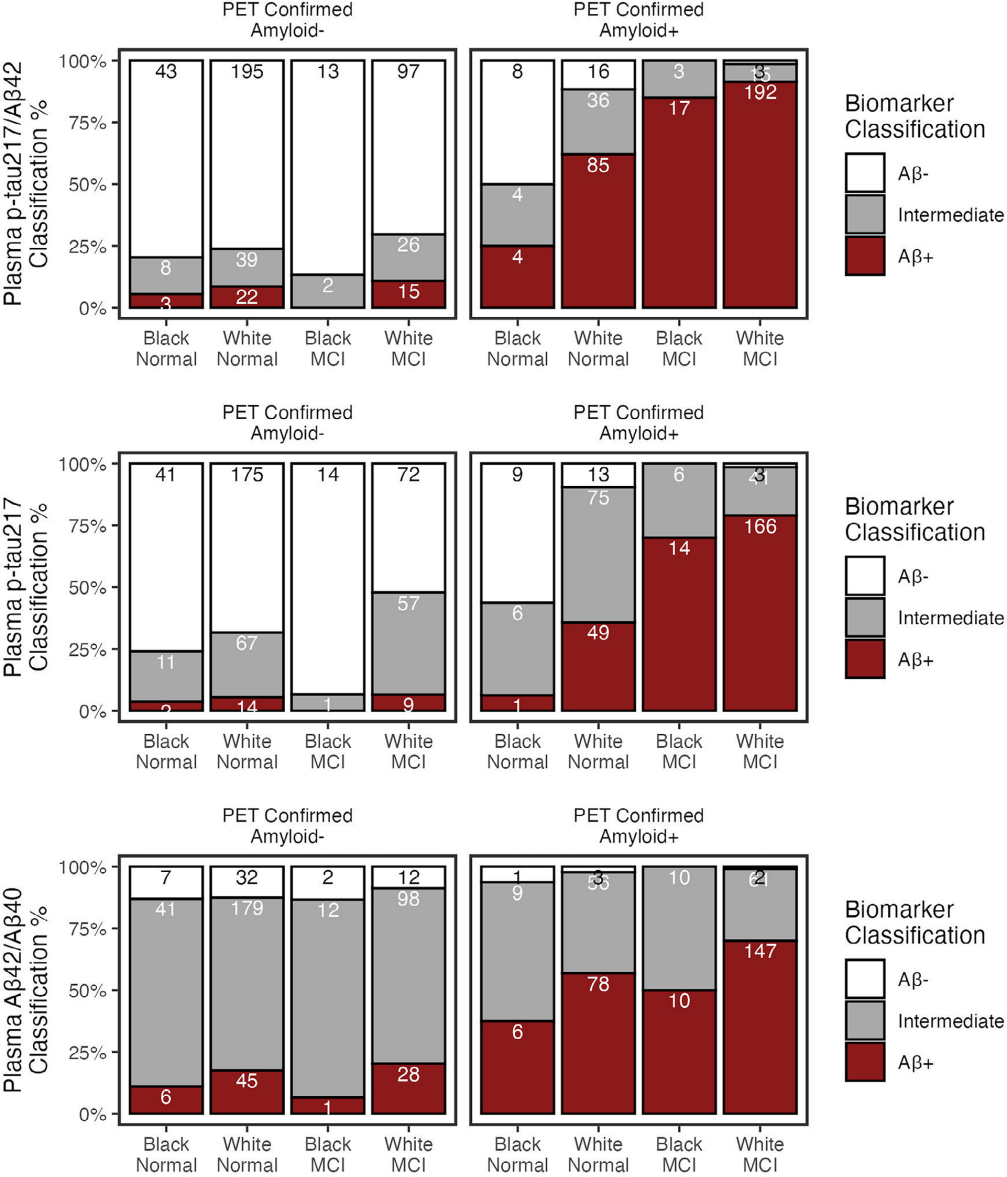
Application of biomarker thresholds in ADNI test set (*n* = 846). Classification performance of biomarker thresholds by race in ADNI. Barplots show percentages (%) of biomarker classifications as amyloid negative (white), intermediate (gray), or amyloid positive (red). Classifications are split by pWhite (*n* = 741) and pBlack (*n* = 105), and by cognitively normal (*n* = 463) and MCI (*n* = 383). Left panels are amyloid negative; right panels are amyloid positive. Count for each classification are labeled in bars.

We applied derived‐biomarker thresholds in the UPenn training set (Table 4), and validated biomarker thresholds in the ADNI test set (Table 4; Figure 4; Figure S4). In ADNI, plasma p‐tau217/Aβ42 had excellent classification accuracy in pWhite (0.91) and pBlack (0.88). Total errors did not differ by race (*p *= 0.33), although we note 0.72 sensitivity and false negative errors in Normal pBlack (Figure 4). Plasma p‐tau217/Aβ42 also had the smallest proportion of intermediate/unclassified values (range = 0.09–0.16).

**TABLE 4 alz70469-tbl-0004:** Application of biomarker thresholds in training (*n* = 289) and test sets (*n* = 846).

A. Performance	Biomarker	Proportion intermediate	Accuracy	95% CI	Sens	Spec	PPV	NPV
UPenn; All	p‐tau217/Aβ42	0.09	0.95	0.91–0.97	0.94	0.95	0.93	0.96
UPenn; White	p‐tau217/Aβ42	0.10	0.94	0.90–0.97	0.94	0.94	0.93	0.95
UPenn; Black	p‐tau217/Aβ42	0.09	0.96	0.88–0.99	0.95	0.96	0.90	0.98
ADNI; All	p‐tau217/Aβ42	0.16	0.91	0.88–0.93	0.92	0.90	0.88	0.93
ADNI; White	p‐tau217/Aβ42	0.16	0.91	0.89–0.93	0.94	0.89	0.88	0.94
ADNI; Black	p‐tau217/Aβ42	0.16	0.88	0.79–0.94	**0.72**	0.95	0.88	0.88
UPenn; All	p‐tau217	0.22	0.94	0.90–0.97	0.93	0.94	0.91	0.96
UPenn; White	p‐tau217	0.23	0.94	0.89–0.97	0.93	0.94	0.93	0.94
UPenn; Black	p‐tau217	0.19	0.94	0.85–0.98	0.93	0.94	0.82	0.98
ADNI; All	p‐tau217	0.31	0.91	0.89–0.94	0.90	0.92	0.90	0.92
ADNI; White	p‐tau217	0.32	0.92	0.90–0.94	0.93	0.91	0.90	0.94
ADNI; Black	p‐tau217	0.23	0.86	0.77–0.93	**0.62**	0.96	0.88	0.86
UPenn; All	Aβ42/Aβ40	0.55	0.89	0.83–0.94	0.87	0.90	0.84	0.93
UPenn; White	Aβ42/Aβ40	0.62	0.89	0.80–0.95	0.91	0.87	0.84	0.93
UPenn; Black	Aβ42/Aβ40	0.38	0.90	0.78–0.97	**0.75**	0.95	0.82	0.92
ADNI; All	Aβ42/Aβ40	0.55	**0.77**	0.73–0.81	0.98	**0.40**	**0.75**	0.90
ADNI; White	Aβ42/Aβ40	0.53	**0.78**	0.73–0.82	0.98	**0.38**	**0.76**	0.90
ADNI; Black	Aβ42/Aβ40	0.69	**0.76**	0.58–0.89	0.94	**0.56**	**0.70**	0.90

Derived from UPenn training set, thresholds determined “Aβ+”, “Aβ−”, and “Intermediate” classifications. Thresholds were applied in both UPenn (training; 209 pWhite and 80 pBlack) and ADNI (test; 741 pWhite and 105 pBlack) datasets. Performance metrics (accuracy, accuracy 95% confidence interval [95%CI], sensitivity [Sens], specificity [Spec], positive predictive value [PPV], negative predictive value [NPV]) are based on subjects classified as Aβ+ or Aβ−; performance values < 0.80 are in red. Intermediate cases were excluded from performance calculations (accuracy, 95% CI, Sens, Spec, PPV, NPV).

Plasma p‐tau217 had the next best overall performance (accuracy range = 0.86–0.94; intermediate range = 0.19–0.32); error rate was not significantly worse in pBlack than pWhite (OR = 1.86, 95% CI = 0.82–3.92, *p *= 0.089). Plasma Aβ42/Aβ40 had the lowest accuracy (range = 0.76–0.90) and highest proportion intermediate (range = 0.38–0.69); its classification did not differ by race (*p *= 0.83).

Classification results were similar in matched samples (Figure S5).

We tested for racial differences in biomarkers in the matched ADNI subsample (*n* = 210; Table S6; Figures S6 and S7). Models accounting for amyloid status and covariates (eTable 7) showed that lower p‐tau217 (β = −0.24, 95% CI = −0.45 to −0.025, *p *= 0.029) with small effect (η^2^ *_G _* = 0.034) did not survive correction for multiple comparisons (Bonferroni‐*p *= 0.086). Neither plasma p‐tau217/Aβ42 (*p *= 0.11) nor Aβ42/Aβ40 differed significantly by race (*p *= 0.39). In addition, we note that higher creatinine associated with higher p‐tau217 (β = 0.75, 95% CI = 0.26–1.2, *p *= 0.0031; Bonferroni‐*p *= 0.0094) with medium effect (η^2^ *_G _* = 0.062), Aβ42 (β = 0.38, 95% CI = 0.25–0.5, *p *= 2.8e‐08; Bonferroni‐*p *= 8.3e‐08) with large effect (η^2^ *_G _* = 0.20), and Aβ40 (β = 0.31, 95% CI = 0.2–0.42, *p *= 9.3e‐08; Bonferroni‐*p *= 2.8e‐07) with large effect (η^2^ *_G _* = 0.19), but not p‐tau217/Aβ42 (*p *= 0.15) or Aβ42/Aβ40 (*p *= 0.12).

Race × amyloid PET Centiloid interactions (Table S8) were not significant for p‐tau217/Aβ42 (*p *= 0.99), p‐tau217 (*p *= 0.99), or Aβ42/Aβ40 (*p *= 0.54).

#### 3.3.1 Post hoc tests

Because plasma p‐tau217/Aβ42 had the highest overall accuracy in ADNI, Mann–Whitney–Wilcoxon and Fisher's exact tests interrogated which patient factors might influence p‐tau217/Aβ42 accuracy. Compared to correctly classified cases (*n* = 646; normal cognition: *n* = 327 [51%]; female: *n* = 330 [51%]; age: median = 73, IQR = 11; BMI: median = 26.7, IQR = 5.9; creatinine: median = 0.9, IQR = 0.3), false negatives (*n* = 27) were more likely to be cognitively normal (*n* = 24 [89%]; OR = 7.79, 95% CI = 2.33–40.81, *p *= 5.1e‐05), female (*n* = 21 [78%]; OR = 3.35, 95% CI = 1.2810.27, *p *= 0.0093), have higher BMI (28.7, IQR = 8.9; W = 4953, *p *= 0.044), and lower creatinine (0.8, IQR = 0.2; W = 6238.5, *p *= 0.033). False negatives did not differ significantly from correctly classified cases by age (median = 70, IQR = 6.5; *p *= 0.21).

False positives (*n* = 40) did not differ in sex distribution (female *n* = 15 [38%]; *p *= 0.10), cognitive status (normal *n* = 25 [62%]; *p *= 0.19), age (median = 74.5, IQR = 7.2; *p *= 0.53), BMI (median = 26.2, IQR = 7.3; *p *= 0.76), or creatinine (median = 1, IQR = 0.2; *p *= 0.16).

Supplementary ROC analyses tested a linear combination of p‐tau217 + Aβ42/Aβ40 (Tables S9 and S10), which showed comparable/slightly lower accuracy than the p‐tau217/Aβ42 ratio and a higher proportion of Intermediates (Figure S8). We also tested classification performance using a one cut point strategy, which demonstrated lower accuracy than two cut points (Tables S11 and S12).

Finally, Table S13 compared biomarker levels and demographics (cognition, age, BMI, sex, education, *APOE* ε4, and ADI) of the training and test samples. Compared to UPenn pBlack (training set), ADNI pBlack (test set) had significantly lower plasma Aβ42/Aβ40 (W = 2272, *p *= 9.2e‐08), with more MCI (χ^2^(1) = 4.2, *p *= 0.041), female (χ^2^(1) = 5.8, *p *= 0.016), and younger age (W = 3184, *p *= 0.0048). Compared to UPenn pWhite, ADNI pWhite had significantly lower plasma Aβ42/Aβ40 (W = 50600, *p *= 1.9e‐14), higher p‐tau217/Aβ42 (W = 85196, *p *= 0.027), and higher ADI (χ^2^(2) = 17, *p *= 0.00026).

## 4 DISCUSSION

Plasma biomarkers have[Table alz70469-tbl-0003] the[Table alz70469-tbl-0004] potential to democratize access to an AD diagnosis. Demand for biomarker testing is expected to increase, making it essential to validate biomarker performance and robustness across a variety of patient factors, including racial identity and disease stage.^12^, ^14^, ^18^, ^20^, ^21^, ^44^ Here we evaluated plasma p‐tau217/Aβ42, p‐tau217, and Aβ42/Aβ40 to detect AD, and investigated whether classification[Fig alz70469-fig-0004] accuracy differed by race or cognitive status, using amyloid PET data as the gold standard. We advanced this work by robustly matching groups to mitigate sampling biases, and by validating cut points in an independent test set. We found that the plasma p‐tau217/Aβ42 ratio consistently had the highest performance across race and cognition. Although p‐tau217/Aβ42 accuracy was not significantly better than p‐tau217 alone,^45^ p‐tau217/Aβ42 classifications had significantly fewer intermediate cases than either p‐tau217 or Aβ42/Aβ40, potentially improving diagnostic clarity.^46^ Likewise, the ratio of p‐tau217/Aβ42 has the strongest association with amyloid pathology,^4^, ^47^ and may help to mitigate the influence of confounding factors.^39^, ^47^ In sum, the p‐tau217/Aβ42 ratio maximizes classification accuracy while simultaneously minimizing the proportion of intermediate/unclassifiable cases.

Of note, both UPenn and ADNI pBlack skewed younger, female, cognitively normal, with higher BMI and less severe amyloid PET; likewise, these factors also associated with false negative errors and may contribute to a somewhat lower sensitivity of p‐tau217/Aβ42 and p‐tau217 in pBlack than pWhite. To account for potential sampling biases,^15^, ^26^ we rigorously applied propensity score matching across amyloid severity and other factors. Matched analyses demonstrated plasma Aβ42/Aβ40 was higher in UPenn pBlack than pWhite, consistent with other studies,^18^, ^19^ but not reproduced in the ADNI sample. And there were no differences in Aβ42/Aβ40 classification accuracy by race. Although ADNI pBlack had significantly lower Aβ42/Aβ40 than UPenn pBlack, it is somewhat unclear why Aβ42/Aβ40 was higher in matched UPenn pBlack compared to pWhite (driven by amyloid negative, cognitively normal groups); unobserved differences in comorbidities (e.g., diabetes) may contribute to differences across datasets. It is important to note that no biomarkers had altered associations with amyloid PET severity by race (all null interactions), suggesting that the relationship between plasma levels and amyloid burden is consistent by racial identity. Still, we note that racial differences had a small effect size, so larger studies may be needed to detect an interaction. Nonetheless, our overall findings indicate that race alone is not a major factor in plasma biomarker levels.

In models (Tables S3–S5, S7, and S8), we examined other factors that may affect plasma levels, including SSDOH and medical morbidities,^15^, ^47^, ^48^ which can associate with racial identity.^49^, ^50^ ADI was not associated with biomarkers, and future investigations should test if more fine‐grained SSDOH metrics affect plasma biomarkers. By contrast, several medical morbidities were associated with plasma levels. In the UPenn dataset, pBlack had a higher incidence of diabetes than pWhite;^51^ likewise, diabetes was associated with lower plasma Aβ42/Aβ40 and higher Aβ40, corroborated by other studies.^48^ This relationship may be due in part to the overlap between diabetes and kidney disease,^52^ a known factor in plasma biomarker levels.^13^, ^38^ In the ADNI dataset, a subset of individuals had creatinine as a metric of kidney dysfunction. Although creatinine levels did not differ by race, high creatinine was associated with higher plasma p‐tau217, Aβ42, and Aβ40, as observed previously.^38^, ^39^ It is notable that creatinine was not a significant factor for p‐tau217/Aβ42 levels in this study (or Aβ42/Aβ40), further showing that ratios may help to mitigate potential confounds. BMI may also influence plasma levels,^38^ and we found that false‐negative errors tended to have a higher BMI; yet models showed that BMI was not a significant factor for biomarker levels (neither UPenn nor ADNI datasets) after accounting for other covariates. Finally, we found that *APOE* ε4 alleles were associated with plasma biomarker levels.^37^

**Limitations**. Several caveats must be considered when interpreting results. First, additional factors may influence blood biomarker levels that were not available in this study. The incidence of concomitant neural pathologies, such as vascular disease and neuronal α‐synuclein disease (NSD), can also differ by race,^53^, ^54^ and may be an important factor in plasma biomarker levels^55^, ^56^ for future studies to investigate. Second, the majority of pBlack in this study had normal cognition (73%); likewise, the matched sample was 80% cognitively normal participants. Non‐AD factors, such as BMI and renal dysfunction, may have proportionally larger effects on biomarker levels in cognitively normal or amyloid‐negative individuals,^13^, ^38^ compared to when AD pathology is severe.^14^ Likewise here, differences in plasma levels by race were most apparent in amyloid‐negative individuals. Future studies in diverse, cognitively impaired cohorts are needed. Third, due to data availability, covariates were slightly different between UPenn (did not include BMI or creatinine) and ADNI models (did not include diabetes or ADI); however, inclusion/exclusion of these covariates did not alter the main conclusions of this study. Fourth, although we evaluated biomarkers in a racially diverse sample, our data were collected from specialized memory centers.^11^, ^16^ Continued efforts in population‐based recruitment and validation studies to ensure generalizability are essential to the implementation of blood biomarkers in the clinic.

## AUTHOR CONTRIBUTIONS

Katheryn A.Q. Cousins, Leslie M. Shaw, and David A. Wolk had major contributions to the conception of the research question and overall design of the study. Magdalena Korecka, Yang Wan, Amberley Vulaj, Christopher Brown, Thomas F. Tropea, Edward B. Lee, David J. Irwin, David A. Wolk, and Leslie M. Shaw were involved in data acquisition and analysis, including imaging and biofluid analysis. Katheryn A.Q. Cousins performed all statistical analyses and comparisons in this study. Katheryn A.Q. Cousins was involved in the initial manuscript drafting. Katheryn A.Q. Cousins, Leslie M. Shaw, and David A. Wolk were all involved in the manuscript drafting and editing, and all authors have approved the final draft.

## CONFLICT OF INTEREST STATEMENT

E.B.L. has received consulting fees from Lilly and Wavebreak Therapeutics. All other authors have report no conflicts of interest relevant to this study. Any author disclosures are available in the Supporting Information.

## CONSENT STATEMENT

Written informed consent was obtained for both UPenn and ADNI participants according to the Declaration of Helsinki and approved by the Institutional Review Boards.

## DATA ACCESS

K.A.Q.C. and L.M.S. had full access to all the data in the study and take responsibility for the integrity of the data and the accuracy of the data analysis.

## Supporting information



Supporting Information

Supporting Information

## Data Availability

Anonymized data will be shared by a reasonable request from any qualified investigator.
