# Correction to “Comparison of plasma p‐tau217/Aβ42, p‐tau217, and Aβ42/Aβ40 biomarkers by race to detect Alzheimer's disease”

**DOI:** 10.1002/alz.70913

**Published:** 2025-11-06

Cousins KAQ, Korecka M, Wan Y, et al. Comparison of plasma p‐tau217/Aβ42, p‐tau217, and Aβ42/Aβ40 biomarkers by race to detect Alzheimer's disease. *Alzheimers Dement*.2025; 21(8): e70469. doi:10.1002/alz.70469

In the Acknowledgments Section, the text “This work is supported by funding from the NIA (P01‐AG066597, P30‐AG072979, R01‐AG087258), and the UPenn Institute on Aging.” did not include all grant funding for the study. It should have read, “This work is supported by funding from the NIA (P01‐AG066597, P30‐AG072979, R01‐AG087258), the Pennsylvania Department of Health (2019NF4100087335), and the UPenn Institute on Aging.”

We apologize for this error.

