# Supplementary material for: Comparison of plasma p‐tau217/Aβ42, p‐tau217, and Aβ42/Aβ40 biomarkers by race to detect Alzheimer's disease
Source: Alzheimers Dement. 2025 Aug 13;21(8):e70469. doi: 10.1002/alz.70469 (PMC12344574; doi:10.1002/alz.70469)
Supplement: Supplementary file 1 — Supporting Information [file ALZ-21-e70469-s002.pdf]

ICMJE DISCLOSURE FORM

**Date:**	5/8/2025
**Your Name:**	Katheryn Cousins
**Manuscript Title:**	Comparison of plasma biomarkers by race to detect Alzheimer's disease
**Manuscript Number (if known):**	ADJ-D-25-00365

In the interest of transparency, we ask you to disclose all relationships/activities/interests listed below that are related to the content of your manuscript. "Related" means any relation with for-profit or not-for-profit third parties whose interests may be affected by the content of the manuscript. Disclosure represents a commitment to transparency and does not necessarily indicate a bias. If you are in doubt about whether to list a relationship/activity/interest, it is preferable that you do so.

The author's relationships/activities/interests should be defined broadly. For example, if your manuscript pertains to the epidemiology of hypertension, you should declare all relationships with manufacturers of antihypertensive medication, even if that medication is not mentioned in the manuscript.

In item #1 below, report all support for the work reported in this manuscript without time limit. For all other items, the time frame for disclosure is the past 36 months.

		Name all entities with whom you have this relationship or indicate none (add rows as needed)	Specifications/Comments (e.g., if payments were made to you or to your institution)
		**Time frame: Since the initial planning of the work**	
1	All support for the present manuscript (e.g., funding, provision of study materials, medical writing, article processing charges, etc.) **No time limit for this item.**	☐ None	
		National Institutes of Health/National Institute on Aging (R01-AG087258)	
		National Institute on Aging (P30 AG072979)	
			Click the tab key to add additional rows.
		**Time frame: past 36 months**	
2	Grants or contracts from any entity (if not indicated in item #1 above).	☐ **None**	
		AL220035 -- Department of Defense (DOD) Amyotrophic Lateral Sclerosis Research Program (ALSRP)	
3	Royalties or licenses	☒ **None**	

		Name all entities with whom you have this relationship or indicate none (add rows as needed)	Specifications/Comments (e.g., if payments were made to you or to your institution)
**4**	Consulting fees	☒ **None**	
**5**	Payment or honoraria for lectures, presentations, speakers bureaus, manuscript writing or educational events	☒ **None**	
**6**	Payment for expert testimony	☒ **None**	
**7**	Support for attending meetings and/or travel	☒ **None**	
**8**	Patents planned, issued or pending	☒ **None**	
**9**	Participation on a Data Safety Monitoring Board or Advisory Board	☒ **None**	
**10**	Leadership or fiduciary role in other board, society, committee or advocacy group, paid or unpaid	☒ **None**	

		Name all entities with whom you have this relationship or indicate none (add rows as needed)	Specifications/Comments (e.g., if payments were made to you or to your institution)
**11**	Stock or stock options	☒ **None**	
**12**	Receipt of equipment, materials, drugs, medical writing, gifts or other services	☒ **None**	
**13**	Other financial or non-financial interests	☒ **None**	

Please place an "X" next to the following statement to indicate your agreement:

☒ I certify that I have answered every question and have not altered the wording of any of the questions on this form.

ICMJE DISCLOSURE FORM

Date: 3/25/2025

Your Name: Thomas F Tropea

Manuscript Title: Comparison of plasma biomarkers by race to detect Alzheimer's disease

Manuscript Number (if known): ADJ-D-25-00365

In the interest of transparency, we ask you to disclose all relationships/activities/interests listed below that are related to the content of your manuscript. "Related" means any relation with for-profit or not-for-profit third parties whose interests may be affected by the content of the manuscript. Disclosure represents a commitment to transparency and does not necessarily indicate a bias. If you are in doubt about whether to list a relationship/activity/interest, it is preferable that you do so.

The author's relationships/activities/interests should be defined broadly. For example, if your manuscript pertains to the epidemiology of hypertension, you should declare all relationships with manufacturers of antihypertensive medication, even if that medication is not mentioned in the manuscript.

In item #1 below, report all support for the work reported in this manuscript without time limit. For all other items, the time frame for disclosure is the past 36 months.

		Name all entities with whom you have this relationship or indicate none (add rows as needed)	Specifications/Comments (e.g., if payments were made to you or to your institution)
**Time frame: Since the initial planning of the work**			
1	All support for the present manuscript (e.g., funding, provision of study materials, medical writing, article processing charges, etc.) **No time limit for this item.**	☐ **None**	
		NIH-NINDS- K23NS114167	Institution
			Click the tab key to add additional rows.
**Time frame: past 36 months**			
**2**	Grants or contracts from any entity (if not indicated in item #1 above).	☐ **None**	
		NIH-NIA- P30AG072979	Institution
		Michael J Fox Foundation	Institution
		Parkinson Foundation	Institution
		Eli Lilly and Company	Institution
**3**	Royalties or licenses	☒ **None**	

		Name all entities with whom you have this relationship or indicate none (add rows as needed)	Specifications/Comments (e.g., if payments were made to you or to your institution)
**4**	Consulting fees	☐ **None**	
		Bial	Me
**5**	Payment or honoraria for lectures, presentations, speakers bureaus, manuscript writing or educational events	☐ **None**	
		Catalyst Medical Education	Me
**6**	Payment for expert testimony	☒ **None**	
**7**	Support for attending meetings and/or travel	☐ **None**	
		Michael J Fox Foundation	Me
		Parkinson Foundation	Me
**8**	Patents planned, issued or pending	☒ **None**	
**9**	Participation on a Data Safety Monitoring Board or Advisory Board	☒ **None**	
**10**	Leadership or fiduciary role in other board, society, committee or advocacy group, paid or unpaid	☒ **None**	

		Name all entities with whom you have this relationship or indicate none (add rows as needed)	Specifications/Comments (e.g., if payments were made to you or to your institution)
**11**	Stock or stock options	☒ **None**	
**12**	Receipt of equipment, materials, drugs, medical writing, gifts or other services	☒ **None**	
**13**	Other financial or non-financial interests	☒ **None**	

Please place an "X" next to the following statement to indicate your agreement:

☒ I certify that I have answered every question and have not altered the wording of any of the questions on this form.

ICMJE DISCLOSURE FORM

**Date:**	5/8/2025
**Your Name:**	Yang Wan
**Manuscript Title:**	Comparison of plasma biomarkers by race to detect Alzheimer's disease
**Manuscript Number (if known):**	ADJ-D-25-00365

In the interest of transparency, we ask you to disclose all relationships/activities/interests listed below that are related to the content of your manuscript. "Related" means any relation with for-profit or not-for-profit third parties whose interests may be affected by the content of the manuscript. Disclosure represents a commitment to transparency and does not necessarily indicate a bias. If you are in doubt about whether to list a relationship/activity/interest, it is preferable that you do so.

The author's relationships/activities/interests should be defined broadly. For example, if your manuscript pertains to the epidemiology of hypertension, you should declare all relationships with manufacturers of antihypertensive medication, even if that medication is not mentioned in the manuscript.

In item #1 below, report all support for the work reported in this manuscript without time limit. For all other items, the time frame for disclosure is the past 36 months.

		**Name all entities with whom you have this relationship or indicate none (add rows as needed)**	**Specifications/Comments (e.g., if payments were made to you or to your institution)**
**Time frame: Since the initial planning of the work**			
1	All support for the present manuscript (e.g., funding, provision of study materials, medical writing, article processing charges, etc.) **No time limit for this item.**	☐ **None**	
		Biomarker data analysis & sample demographic information process.	
			Click the tab key to add additional rows.
**Time frame: past 36 months**			
**2**	Grants or contracts from any entity (if not indicated in item #1 above).	☒ **None**	
**3**	Royalties or licenses	☒ **None**	

		Name all entities with whom you have this relationship or indicate none (add rows as needed)	Specifications/Comments (e.g., if payments were made to you or to your institution)
**4**	Consulting fees	☒ **None**	
**5**	Payment or honoraria for lectures, presentations, speakers bureaus, manuscript writing or educational events	☒ **None**	
**6**	Payment for expert testimony	☒ **None**	
**7**	Support for attending meetings and/or travel	☒ **None**	
**8**	Patents planned, issued or pending	☒ **None**	
**9**	Participation on a Data Safety Monitoring Board or Advisory Board	☒ **None**	
**10**	Leadership or fiduciary role in other board, society, committee or advocacy group, paid or unpaid	☒ **None**	

		Name all entities with whom you have this relationship or indicate none (add rows as needed)	Specifications/Comments (e.g., if payments were made to you or to your institution)
**11**	Stock or stock options	☒ **None**	
**12**	Receipt of equipment, materials, drugs, medical writing, gifts or other services	☒ **None**	
**13**	Other financial or non-financial interests	☒ **None**	

Please place an "X" next to the following statement to indicate your agreement:

☒ I certify that I have answered every question and have not altered the wording of any of the questions on this form.

ICMJE DISCLOSURE FORM

**Date:**	5/8/2025
**Your Name:**	Magdalena Korecka
**Manuscript Title:**	Comparison of plasma biomarkers by race to detect Alzheimer's disease
**Manuscript Number (if known):**	ADJ-D-25-00365

In the interest of transparency, we ask you to disclose all relationships/activities/interests listed below that are related to the content of your manuscript. "Related" means any relation with for-profit or not-for-profit third parties whose interests may be affected by the content of the manuscript. Disclosure represents a commitment to transparency and does not necessarily indicate a bias. If you are in doubt about whether to list a relationship/activity/interest, it is preferable that you do so.

The author's relationships/activities/interests should be defined broadly. For example, if your manuscript pertains to the epidemiology of hypertension, you should declare all relationships with manufacturers of antihypertensive medication, even if that medication is not mentioned in the manuscript.

In item #1 below, report all support for the work reported in this manuscript without time limit. For all other items, the time frame for disclosure is the past 36 months.

		**Name all entities with whom you have this relationship or indicate none (add rows as needed)**	**Specifications/Comments (e.g., if payments were made to you or to your institution)**
**Time frame: Since the initial planning of the work**			
1	All support for the present manuscript (e.g., funding, provision of study materials, medical writing, article processing charges, etc.) **No time limit for this item.**	☒ None	
			Click the tab key to add additional rows.
**Time frame: past 36 months**			
2	Grants or contracts from any entity (if not indicated in item #1 above).	☒ **None**	
3	Royalties or licenses	☒ **None**	

		Name all entities with whom you have this relationship or indicate none (add rows as needed)	Specifications/Comments (e.g., if payments were made to you or to your institution)
**4**	Consulting fees	☒ **None**	
**5**	Payment or honoraria for lectures, presentations, speakers bureaus, manuscript writing or educational events	☒ **None**	
**6**	Payment for expert testimony	☒ **None**	
**7**	Support for attending meetings and/or travel	☒ **None**	
**8**	Patents planned, issued or pending	☒ **None**	
**9**	Participation on a Data Safety Monitoring Board or Advisory Board	☒ **None**	
**10**	Leadership or fiduciary role in other board, society, committee or advocacy group, paid or unpaid	☒ **None**	

		Name all entities with whom you have this relationship or indicate none (add rows as needed)	Specifications/Comments (e.g., if payments were made to you or to your institution)
**11**	Stock or stock options	☒ **None**	
**12**	Receipt of equipment, materials, drugs, medical writing, gifts or other services	☒ **None**	
**13**	Other financial or non-financial interests	☒ **None**	

Please place an "X" next to the following statement to indicate your agreement:

☒ I certify that I have answered every question and have not altered the wording of any of the questions on this form.

ICMJE DISCLOSURE FORM

**Date:**	5/8/2025
**Your Name:**	Duygu Tosun
**Manuscript Title:**	Comparison of plasma biomarkers by race to detect Alzheimer's disease
**Manuscript Number (if known):**	ADJ-D-25-00365

In the interest of transparency, we ask you to disclose all relationships/activities/interests listed below that are related to the content of your manuscript. "Related" means any relation with for-profit or not-for-profit third parties whose interests may be affected by the content of the manuscript. Disclosure represents a commitment to transparency and does not necessarily indicate a bias. If you are in doubt about whether to list a relationship/activity/interest, it is preferable that you do so.

The author's relationships/activities/interests should be defined broadly. For example, if your manuscript pertains to the epidemiology of hypertension, you should declare all relationships with manufacturers of antihypertensive medication, even if that medication is not mentioned in the manuscript.

In item #1 below, report all support for the work reported in this manuscript without time limit. For all other items, the time frame for disclosure is the past 36 months.

		Name all entities with whom you have this relationship or indicate none (add rows as needed)	Specifications/Comments (e.g., if payments were made to you or to your institution)
**Time frame: Since the initial planning of the work**			
1	All support for the present manuscript (e.g., funding, provision of study materials, medical writing, article processing charges, etc.) **No time limit for this item.**	☐ **None**	
		NIH/NIA	To my institution
			Click the tab key to add additional rows.
**Time frame: past 36 months**			
**2**	Grants or contracts from any entity (if not indicated in item #1 above).	☒ **None**	
**3**	Royalties or licenses	☒ **None**	

		Name all entities with whom you have this relationship or indicate none (add rows as needed)	Specifications/Comments (e.g., if payments were made to you or to your institution)
**4**	Consulting fees	☒ **None**	
**5**	Payment or honoraria for lectures, presentations, speakers bureaus, manuscript writing or educational events	☒ **None**	
**6**	Payment for expert testimony	☒ **None**	
**7**	Support for attending meetings and/or travel	☒ **None**	
**8**	Patents planned, issued or pending	☒ **None**	
**9**	Participation on a Data Safety Monitoring Board or Advisory Board	☒ **None**	
**10**	Leadership or fiduciary role in other board, society, committee or advocacy group, paid or unpaid	☒ **None**	

		Name all entities with whom you have this relationship or indicate none (add rows as needed)	Specifications/Comments (e.g., if payments were made to you or to your institution)
**11**	Stock or stock options	☒ **None**	
**12**	Receipt of equipment, materials, drugs, medical writing, gifts or other services	☒ **None**	
**13**	Other financial or non-financial interests	☒ **None**	

Please place an "X" next to the following statement to indicate your agreement:

☒ I certify that I have answered every question and have not altered the wording of any of the questions on this form.

ICMJE DISCLOSURE FORM

Date: 5/8/2025

Your Name: Edward B. Lee

Manuscript Title: Comparison of plasma biomarkers by race to detect Alzheimer's disease

Manuscript Number (if known): ADJ-D-25-00365

In the interest of transparency, we ask you to disclose all relationships/activities/interests listed below that are related to the content of your manuscript. "Related" means any relation with for-profit or not-for-profit third parties whose interests may be affected by the content of the manuscript. Disclosure represents a commitment to transparency and does not necessarily indicate a bias. If you are in doubt about whether to list a relationship/activity/interest, it is preferable that you do so.

The author's relationships/activities/interests should be defined broadly. For example, if your manuscript pertains to the epidemiology of hypertension, you should declare all relationships with manufacturers of antihypertensive medication, even if that medication is not mentioned in the manuscript.

In item #1 below, report all support for the work reported in this manuscript without time limit. For all other items, the time frame for disclosure is the past 36 months.

		Name all entities with whom you have this relationship or indicate none (add rows as needed)	Specifications/Comments (e.g., if payments were made to you or to your institution)
**Time frame: Since the initial planning of the work**			
1	All support for the present manuscript (e.g., funding, provision of study materials, medical writing, article processing charges, etc.) **No time limit for this item.**	☐ **None**	
		NIH grants	
			Click the tab key to add additional rows.
**Time frame: past 36 months**			
**2**	Grants or contracts from any entity (if not indicated in item #1 above).	☐ **None**	
		Delaware Community Foundation	
		NIH grants	
**3**	Royalties or licenses	☒ **None**	

		Name all entities with whom you have this relationship or indicate none (add rows as needed)	Specifications/Comments (e.g., if payments were made to you or to your institution)
**4**	Consulting fees	☐ **None**	
		Wavebreak Therapeutics	
		Eli Lilly	
**5**	Payment or honoraria for lectures, presentations, speakers bureaus, manuscript writing or educational events	☐ **None**	
		Multiple non-profit and/or academic institutions/organizations for research lectures (unrelated to this study)	
**6**	Payment for expert testimony	☒ **None**	
**7**	Support for attending meetings and/or travel	☐ **None**	
		Travel to Tau 2025 meeting (Rainwater Charitable Foundation)	
		Travel to give lectures from multiple non-profit and/or academic institutions/organizations (unrelated to this study)	
**8**	Patents planned, issued or pending	☐ **None**	
		Patent pending on VCP activators (unrelated to this study)	
**9**	Participation on a Data Safety Monitoring Board or Advisory Board	☐ **None**	
		UTSA Alzheimer's Disease Research Center External Advisory Board	American Board of Pathology Test Development Advisory Committee
		University of Florida Alzheimer's Disease Research Center External Advisory Board	Study sections (ADDF, AFTD, NIH)
		Rainwater Prize Selection Committee	
**10**	Leadership or fiduciary role in other board, society, committee or advocacy group, paid or unpaid	☐ **None**	
		AANP Executive Council	

		Name all entities with whom you have this relationship or indicate none (add rows as needed)	Specifications/Comments (e.g., if payments were made to you or to your institution)
**11**	Stock or stock options	☒ **None**	
**12**	Receipt of equipment, materials, drugs, medical writing, gifts or other services	☒ **None**	
**13**	Other financial or non-financial interests	☒ **None**	

Please place an "X" next to the following statement to indicate your agreement:

☒ I certify that I have answered every question and have not altered the wording of any of the questions on this form.

ICMJE DISCLOSURE FORM

**Date:**	5/8/2025
**Your Name:**	David A. Wolk
**Manuscript Title:**	Comparison of plasma biomarkers by race to detect Alzheimer's disease
**Manuscript Number (if known):**	ADJ-D-25-00365

In the interest of transparency, we ask you to disclose all relationships/activities/interests listed below that are related to the content of your manuscript. "Related" means any relation with for-profit or not-for-profit third parties whose interests may be affected by the content of the manuscript. Disclosure represents a commitment to transparency and does not necessarily indicate a bias. If you are in doubt about whether to list a relationship/activity/interest, it is preferable that you do so.

The author's relationships/activities/interests should be defined broadly. For example, if your manuscript pertains to the epidemiology of hypertension, you should declare all relationships with manufacturers of antihypertensive medication, even if that medication is not mentioned in the manuscript.

In item #1 below, report all support for the work reported in this manuscript without time limit. For all other items, the time frame for disclosure is the past 36 months.

		**Name all entities with whom you have this relationship or indicate none (add rows as needed)**	**Specifications/Comments (e.g., if payments were made to you or to your institution)**
**Time frame: Since the initial planning of the work**			
**1**	All support for the present manuscript (e.g., funding, provision of study materials, medical writing, article processing charges, etc.) **No time limit for this item.**	☐ **None**	
		NIH	Payments to my institution
			Click the tab key to add additional rows.
**Time frame: past 36 months**			
**2**	Grants or contracts from any entity (if not indicated in item #1 above).	☐ **None**	
		NIH	Payments to my institution
		Biogen	Payments to my institution
**3**	Royalties or licenses	☒ **None**	

		Name all entities with whom you have this relationship or indicate none (add rows as needed)	Specifications/Comments (e.g., if payments were made to you or to your institution)
**4**	Consulting fees	☐ **None**	
		Eli Lilly	Payments made to me
		Beckman Coulter	Payments made to me
**5**	Payment or honoraria for lectures, presentations, speakers bureaus, manuscript writing or educational events	☐ **None**	
		Eli Lilly CME	Payments made to me
		Efficient CME	Payments made to me
**6**	Payment for expert testimony	☒ **None**	
**7**	Support for attending meetings and/or travel	☐ **None**	
		Alzheimer's Association	
**8**	Patents planned, issued or pending	☒ **None**	
**9**	Participation on a Data Safety Monitoring Board or Advisory Board	☐ **None**	
		GSK	Payments made to me
**10**	Leadership or fiduciary role in other board, society, committee or advocacy group, paid or unpaid	☒ **None**	

		Name all entities with whom you have this relationship or indicate none (add rows as needed)	Specifications/Comments (e.g., if payments were made to you or to your institution)
**11**	Stock or stock options	☒ **None**	
**12**	Receipt of equipment, materials, drugs, medical writing, gifts or other services	☒ **None**	
**13**	Other financial or non-financial interests	☒ **None**	

Please place an "X" next to the following statement to indicate your agreement:

☒ I certify that I have answered every question and have not altered the wording of any of the questions on this form.

ICMJE DISCLOSURE FORM

Date

Your Name: David Irwin

Manuscript Title: Comparison of plasma biomarkers by race to detect Alzheimer's disease

Manuscript Number (if known): ADJ-D-25-00365

In the interest of transparency, we ask you to disclose all relationships/activities/interests listed below that are related to the content of your manuscript. "Related" means any relation with for-profit or not-for-profit third parties whose interests may be affected by the content of the manuscript. Disclosure represents a commitment to transparency and does not necessarily indicate a bias. If you are in doubt about whether to list a relationship/activity/interest, it is preferable that you do so.

The author's relationships/activities/interests should be defined broadly. For example, if your manuscript pertains to the epidemiology of hypertension, you should declare all relationships with manufacturers of antihypertensive medication, even if that medication is not mentioned in the manuscript.

In item #1 below, report all support for the work reported in this manuscript without time limit. For all other items, the time frame for disclosure is the past 36 months.

		**Name all entities with whom you have this relationship or indicate none (add rows as needed)**	**Specifications/Comments (e.g., if payments were made to you or to your institution)**
**Time frame: Since the initial planning of the work**			
**1**	All support for the present manuscript (e.g., funding, provision of study materials, medical writing, article processing charges, etc.) **No time limit for this item.**	☐ **None**	
		NIH, Penn IOA	Research funding to institution
			Click the Fab key to add additional rows.
**Time frame: past 36 months**			
**2**	Grants or contracts from any entity (if not indicated in item #1 above).	☐ **None**	
		Denali Therapeutics, Alector, Passage Bio, Prevail,	Clinical trial research funding to institution
**3**	Royalties or licenses	☒ **None**	

		Name all entities with whom you have this relationship or indicate none (add rows as needed)	Specifications/Comments (e.g., if payments were made to you or to your institution)
**4**	Consulting fees	☒ **None**	
**5**	Payment or honoraria for lectures, presentations, speakers bureaus, manuscript writing or educational events	☒ **None**	
**6**	Payment for expert testimony	☒ **None**	
**7**	Support for attending meetings and/or travel	☒ **None**	
**8**	Patents planned, issued or pending	☒ **None**	
**9**	Participation on a Data Safety Monitoring Board or Advisory Board	☒ **None**	
**10**	Leadership or fiduciary role in other board, society, committee or advocacy group, paid or unpaid	☐ **None**	
		LBDA Scientific Advisory Board	unpaid
		AFTD Medical Advisory council	unpaid

		Name all entities with whom you have this relationship or indicate none (add rows as needed)	Specifications/Comments (e.g., if payments were made to you or to your institution)
**11**	Stock or stock options	☒ **None**	
**12**	Receipt of equipment, materials, drugs, medical writing, gifts or other services	☒ **None**	
**13**	Other financial or non-financial interests	☒ **None**	

Please place an "X" next to the following statement to indicate your agreement:

☒ I certify that I have answered every question and have not altered the wording of any of the questions on this form.

ICMJE DISCLOSURE FORM

Date: 5/9/2025

Your Name: Christopher A Brown

Manuscript Title: Comparison of plasma biomarkers by race to detect Alzheimer's disease

Manuscript Number (if known): ADJ-D-25-00365

In the interest of transparency, we ask you to disclose all relationships/activities/interests listed below that are related to the content of your manuscript. "Related" means any relation with for-profit or not-for-profit third parties whose interests may be affected by the content of the manuscript. Disclosure represents a commitment to transparency and does not necessarily indicate a bias. If you are in doubt about whether to list a relationship/activity/interest, it is preferable that you do so.

The author's relationships/activities/interests should be defined broadly. For example, if your manuscript pertains to the epidemiology of hypertension, you should declare all relationships with manufacturers of antihypertensive medication, even if that medication is not mentioned in the manuscript.

In item #1 below, report all support for the work reported in this manuscript without time limit. For all other items, the time frame for disclosure is the past 36 months.

		**Name all entities with whom you have this relationship or indicate none (add rows as needed)**	**Specifications/Comments (e.g., if payments were made to you or to your institution)**
**Time frame: Since the initial planning of the work**			
1	All support for the present manuscript (e.g., funding, provision of study materials, medical writing, article processing charges, etc.) **No time limit for this item.**	☐ **None**	
		NIH	Paid to my institution
		Alzheimer's Association	Paid to my institution
			lick the tab key to add additional rows.
**Time frame: past 36 months**			
2	Grants or contracts from any entity (if not indicated in item #1 above).	☒ **None**	
3	Royalties or licenses	☒ **None**	

		Name all entities with whom you have this relationship or indicate none (add rows as needed)	Specifications/Comments (e.g., if payments were made to you or to your institution)
**4**	Consulting fees	☒ **None**	
**5**	Payment or honoraria for lectures, presentations, speakers bureaus, manuscript writing or educational events	☒ **None**	
**6**	Payment for expert testimony	☒ **None**	
**7**	Support for attending meetings and/or travel	☒ **None**	
**8**	Patents planned, issued or pending	☒ **None**	
**9**	Participation on a Data Safety Monitoring Board or Advisory Board	☒ **None**	
**10**	Leadership or fiduciary role in other board, society, committee or advocacy group, paid or unpaid	☒ **None**	

		Name all entities with whom you have this relationship or indicate none (add rows as needed)	Specifications/Comments (e.g., if payments were made to you or to your institution)
**11**	Stock or stock options	☒ **None**	
**12**	Receipt of equipment, materials, drugs, medical writing, gifts or other services	☒ **None**	
**13**	Other financial or non-financial interests	☒ **None**	

Please place an "X" next to the following statement to indicate your agreement:

☒ I certify that I have answered every question and have not altered the wording of any of the questions on this form.

ICMJE DISCLOSURE FORM

**Date:**	5/8/2025
**Your Name:**	Amberley Vulaj
**Manuscript Title:**	Comparison of plasma biomarkers by race to detect Alzheimer's disease
**Manuscript Number (if known):**	ADJ-D-25-00365

In the interest of transparency, we ask you to disclose all relationships/activities/interests listed below that are related to the content of your manuscript. "Related" means any relation with for-profit or not-for-profit third parties whose interests may be affected by the content of the manuscript. Disclosure represents a commitment to transparency and does not necessarily indicate a bias. If you are in doubt about whether to list a relationship/activity/interest, it is preferable that you do so.

The author's relationships/activities/interests should be defined broadly. For example, if your manuscript pertains to the epidemiology of hypertension, you should declare all relationships with manufacturers of antihypertensive medication, even if that medication is not mentioned in the manuscript.

In item #1 below, report all support for the work reported in this manuscript without time limit. For all other items, the time frame for disclosure is the past 36 months.

		**Name all entities with whom you have this relationship or indicate none (add rows as needed)**	**Specifications/Comments (e.g., if payments were made to you or to your institution)**
		Time frame: Since the initial planning of the work	
1	All support for the present manuscript (e.g., funding, provision of study materials, medical writing, article processing charges, etc.) **No time limit for this item.**	☒ None	
			Click the tab key to add additional rows.
		Time frame: past 36 months	
**2**	Grants or contracts from any entity (if not indicated in item #1 above).	☒ **None**	
**3**	Royalties or licenses	☒ **None**	

		Name all entities with whom you have this relationship or indicate none (add rows as needed)	Specifications/Comments (e.g., if payments were made to you or to your institution)
**4**	Consulting fees	☒ **None**	
**5**	Payment or honoraria for lectures, presentations, speakers bureaus, manuscript writing or educational events	☒ **None**	
**6**	Payment for expert testimony	☒ **None**	
**7**	Support for attending meetings and/or travel	☒ **None**	
**8**	Patents planned, issued or pending	☒ **None**	
**9**	Participation on a Data Safety Monitoring Board or Advisory Board	☒ **None**	
**10**	Leadership or fiduciary role in other board, society, committee or advocacy group, paid or unpaid	☒ **None**	

		Name all entities with whom you have this relationship or indicate none (add rows as needed)	Specifications/Comments (e.g., if payments were made to you or to your institution)
**11**	Stock or stock options	☒ **None**	
**12**	Receipt of equipment, materials, drugs, medical writing, gifts or other services	☒ **None**	
**13**	Other financial or non-financial interests	☒ **None**	

Please place an "X" next to the following statement to indicate your agreement:

☒ I certify that I have answered every question and have not altered the wording of any of the questions on this form.

ICMJE DISCLOSURE FORM

**Date:**	
**Your Name:**	The Alzheimer's Disease Neuroimaging Initiative (ADNI)
**Manuscript Title:**	Comparison of plasma biomarkers by race to detect Alzheimer's disease
**Manuscript Number (if known):**	ADJ-D-25-00365

In the interest of transparency, we ask you to disclose all relationships/activities/interests listed below that are related to the content of your manuscript. "Related" means any relation with for-profit or not-for-profit third parties whose interests may be affected by the content of the manuscript. Disclosure represents a commitment to transparency and does not necessarily indicate a bias. If you are in doubt about whether to list a relationship/activity/interest, it is preferable that you do so.

The author's relationships/activities/interests should be defined broadly. For example, if your manuscript pertains to the epidemiology of hypertension, you should declare all relationships with manufacturers of antihypertensive medication, even if that medication is not mentioned in the manuscript.

In item #1 below, report all support for the work reported in this manuscript without time limit. For all other items, the time frame for disclosure is the past 36 months.

		Name all entities with whom you have this relationship or indicate none (add rows as needed)	Specifications/Comments (e.g., if payments were made to you or to your institution)
**Time frame: Since the initial planning of the work**			
1	All support for the present manuscript (e.g., funding, provision of study materials, medical writing, article processing charges, etc.) **No time limit for this item.**	☐ **None**	
		NIH Grant: 2 U19 AG024904.16	Payments were made to my institution.
**Time frame: past 36 months**			
2	Grants or contracts from any entity (if not indicated in item #1 above).	☐ **None**	
		NIH Grant: 5U2CAG060426-04	Payments were made to my institution.
		NIH Grant: 5R01AG058676-02	Payments were made to my institution.
		NIH Grant: 1RF1AG059009-01	Payments were made to my institution.
		NIH Grant: R33 AG062867	Payments were made to my institution.
		NIH Grant: 1R01NS119651-01	Payments were made to my institution.
		NIH Grant: RF1AG062196	Payments were made to my institution.
		NIH Grant: R56AG075744-01A1	Payments were made to my institution.
		Additional support from Department of Defense (DOD)	Payments were made to my institution.
		Additional support from: California Department of Public Health (CDPH)	Payments were made to my institution.
		Additional support from: Siemens	Payments were made to my institution.
		Additional support from: Biogen	Payments were made to my institution.
		Additional support from: Hillblom Foundation	Payments were made to my institution.

		Name all entities with whom you have this relationship or indicate none (add rows as needed)	Specifications/Comments (e.g., if payments were made to you or to your institution)
		Additional support from: Alzheimer's Association	Payments were made to my institution.
		Additional support from: Johnson & Johnson	Payments were made to my institution.
		Additional support from: Kevin and Connie Shanahan	Payments were made to my institution.
		Additional support from: GE	Payments were made to my institution.
		Additional support from: VUmc	Payments were made to my institution.
		Additional support from: Australian Catholic University (HBI-BHR)	Payments were made to my institution.
		Additional support from: The Stroke Foundation	Payments were made to my institution.
		Additional support from: Veterans Administration	Payments were made to my institution.
3	Royalties or licenses	☒ **None**	
4	Consulting fees	☐ **None**	
		Boxer Capital	Payment was made directly to me.
		Cerecin	Payment was made directly to me.
		Clario/BioClinica	Payment was made directly to me.
		Dementia Society of Japan	Payment was made directly to me.
		Eisai	Payment was made directly to me.
		Guidepoint	Payment was made directly to me.
		Health and Wellness Partners	Payment was made directly to me.
		Indiana U.	Payment was made directly to me.
		LCN Consulting	Payment was made directly to me.
		Merck Sharp & Dohme Corp.	Payment was made directly to me.
		Duke U.	Payment was made directly to me.
		Prova Education	Payment was made directly to me.
		T3D Therapeutics	Payment was made directly to me.
		University of Southern CA (USC)	Payment was made directly to me.
		WebMD	Payment was made directly to me.
		MEDA Corp.	Payment was made directly to me.
5	Payment or honoraria for lectures, presentations, speakers bureaus, manuscript writing or educational events	☐ **None**	
		China Association for Alzheimer's Disease (CAAD)	Payment was made directly to me.
		Taipei Medical University	Payment was made directly to me.
		Cleveland Clinic	Payment was made directly to me.
		AD/PD Congress	Payment was made directly to me.
		Foundation of Learning; Health Society (Japan)	Payment was made directly to me.
		INSPIRE Project; U. Toulouse	Payment was made directly to me.
		Japan Society for Dementia Research	Payment was made directly to me.
		Korean Dementia Society	Payment was made directly to me.
		Merck Sharp & Dohme Corp.,	Payment was made directly to me.
		National Center for Geriatrics and Gerontology (NCGG; Japan	Payment was made directly to me.
		University of Southern California (USC)	Payment was made directly to me.

		Name all entities with whom you have this relationship or indicate none (add rows as needed)	Specifications/Comments (e.g., if payments were made to you or to your institution)
**6**	Payment for expert testimony	☒ **None**	
**7**	Support for attending meetings and/or travel	☐ **None**	
		AD/PD Congress	Payment was made either directly to the travel accommodations provider, or reimbursed to me.
		Cleveland Clinic	Payment was made either directly to the travel accommodations provider, or reimbursed to me.
		CTAD Congress	Payment was made either directly to the travel accommodations provider, or reimbursed to me.
		Foundation of Learning; Health Society (Japan)	Payment was made either directly to the travel accommodations provider, or reimbursed to me.
		INSPIRE Project; U. Toulouse	Payment was made either directly to the travel accommodations provider, or reimbursed to me.
		Japan Society for Dementia Research	Payment was made either directly to the travel accommodations provider, or reimbursed to me.
		Korean Dementia Society	Payment was made either directly to the travel accommodations provider, or reimbursed to me.
		Merck Sharp & Dohme Corp.,	Payment was made either directly to the travel accommodations provider, or reimbursed to me.
		National Center for Geriatrics and Gerontology (NCGG; Japan	Payment was made either directly to the travel accommodations provider, or reimbursed to me.
		University of Southern California (USC)	Payment was made either directly to the travel accommodations provider, or reimbursed to me.
**8**	Patents planned, issued or pending	☒ **None**	
**9**	Participation on a Data Safety Monitoring Board or Advisory Board	☐ **None**	
		ADNI Scientific Advisory Board	Leadership
		UCSF Committee for Human Research	Committee Member
**10**	Leadership or fiduciary role in other board, society, committee or advocacy group, paid or unpaid	☐ **None**	
		UCSF Inclusion Diversity Equity & Awareness Committee	Leadership
		Diversity Task Force of the Alzheimer's Disease Neuroimaging Initiative (ADNI)	Leadership

		Name all entities with whom you have this relationship or indicate none (add rows as needed)	Specifications/Comments (e.g., if payments were made to you or to your institution)
**11**	Stock or stock options	☐ **None**	
		Alzeca	Stock options held.
		Alzheon, Inc.	Stock options held.
		ALZPath	Stock options held.
		Anven	Stock options held.
**12**	Receipt of equipment, materials, drugs, medical writing, gifts or other services	☒ **None**	
**13**	Other financial or non-financial interests	☒ **None**	

Please place an "X" next to the following statement to indicate your agreement:

☒ I certify that I have answered every question and have not altered the wording of any of the questions on this form.

ICMJE DISCLOSURE FORM

**Date:**	5/9/2025
**Your Name:**	Ozioma C. Okonkwo
**Manuscript Title:**	Comparison of plasma p-tau217/Aβ42, p-tau217, and Aβ42/Aβ40 biomarkers by race to detect Alzheimer's disease
**Manuscript Number (if known):**	ADJ-D-25-00365

In the interest of transparency, we ask you to disclose all relationships/activities/interests listed below that are related to the content of your manuscript. "Related" means any relation with for-profit or not-for-profit third parties whose interests may be affected by the content of the manuscript. Disclosure represents a commitment to transparency and does not necessarily indicate a bias. If you are in doubt about whether to list a relationship/activity/interest, it is preferable that you do so.

The author's relationships/activities/interests should be defined broadly. For example, if your manuscript pertains to the epidemiology of hypertension, you should declare all relationships with manufacturers of antihypertensive medication, even if that medication is not mentioned in the manuscript.

In item #1 below, report all support for the work reported in this manuscript without time limit. For all other items, the time frame for disclosure is the past 36 months.

		Name all entities with whom you have this relationship or indicate none (add rows as needed)	Specifications/Comments (e.g., if payments were made to you or to your institution)
**Time frame: Since the initial planning of the work**			
1	All support for the present manuscript (e.g., funding, provision of study materials, medical writing, article processing charges, etc.) **No time limit for this item.**	☐ **None**	
		NIH	Grants to my institution
			Click the tab key to add additional rows.
**Time frame: past 36 months**			
2	Grants or contracts from any entity (if not indicated in item #1 above).	☒ **None**	
3	Royalties or licenses	☒ **None**	

		Name all entities with whom you have this relationship or indicate none (add rows as needed)	Specifications/Comments (e.g., if payments were made to you or to your institution)
**4**	Consulting fees	☒ **None**	
**5**	Payment or honoraria for lectures, presentations, speakers bureaus, manuscript writing or educational events	☒ **None**	
**6**	Payment for expert testimony	☒ **None**	
**7**	Support for attending meetings and/or travel	☒ **None**	
**8**	Patents planned, issued or pending	☒ **None**	
**9**	Participation on a Data Safety Monitoring Board or Advisory Board	☒ **None**	
**10**	Leadership or fiduciary role in other board, society, committee or advocacy group, paid or unpaid	☐ **None**	
		International Neuropsychological Society	Treasurer

		Name all entities with whom you have this relationship or indicate none (add rows as needed)	Specifications/Comments (e.g., if payments were made to you or to your institution)
**11**	Stock or stock options	☒ **None**	
**12**	Receipt of equipment, materials, drugs, medical writing, gifts or other services	☒ **None**	
**13**	Other financial or non-financial interests	☒ **None**	

Please place an "X" next to the following statement to indicate your agreement:

☒ I certify that I have answered every question and have not altered the wording of any of the questions on this form.

ICMJE DISCLOSURE FORM

Date: Click or tap to enter a date.

Your Name: Leslie Shaw

Manuscript Title: Comparison of plasma biomarkers by race to detect Alzheimer's disease

Manuscript Number (if known): ADJ-D-25-00365

In the interest of transparency, we ask you to disclose all relationships/activities/interests listed below that are related to the content of your manuscript. "Related" means any relation with for-profit or not-for-profit third parties whose interests may be affected by the content of the manuscript. Disclosure represents a commitment to transparency and does not necessarily indicate a bias. If you are in doubt about whether to list a relationship/activity/interest, it is preferable that you do so.

The author's relationships/activities/interests should be defined broadly. For example, if your manuscript pertains to the epidemiology of hypertension, you should declare all relationships with manufacturers of antihypertensive medication, even if that medication is not mentioned in the manuscript.

In item #1 below, report all support for the work reported in this manuscript without time limit. For all other items, the time frame for disclosure is the past 36 months.

		Name all entities with whom you have this relationship or indicate none (add rows as needed)	Specifications/Comments (e.g., if payments were made to you or to your institution)
		**Time frame: Since the initial planning of the work**	
1	All support for the present manuscript (e.g., funding, provision of study materials, medical writing, article processing charges, etc.) **No time limit for this item.**	☐ **None**	
		NIA/NIH: P30 AG072979; U19 AG024904;	Institution
			Click the tab key to add additional rows.
		**Time frame: past 36 months**	
2	Grants or contracts from any entity (if not indicated in item #1 above).	☐ **None**	
		NIA/NIH: P30 AG072979; U19 AG024904; R01 AG067505	Institution
		DOD: W81XWH2211081-B	
3	Royalties or licenses	☒ **None**	

		Name all entities with whom you have this relationship or indicate none (add rows as needed)	Specifications/Comments (e.g., if payments were made to you or to your institution)
**4**	Consulting fees	☐ **None**	
		Biogen; Roche	To me
**5**	Payment or honoraria for lectures, presentations, speakers bureaus, manuscript writing or educational events	☐ **None**	
		Biogen; Roche	To me
**6**	Payment for expert testimony	☒ **None**	
**7**	Support for attending meetings and/or travel	☒ **None**	
**8**	Patents planned, issued or pending	☒ **None**	
**9**	Participation on a Data Safety Monitoring Board or Advisory Board	☒ **None**	
**10**	Leadership or fiduciary role in other board, society, committee or advocacy group, paid or unpaid	☒ **None**	

		Name all entities with whom you have this relationship or indicate none (add rows as needed)	Specifications/Comments (e.g., if payments were made to you or to your institution)
**11**	Stock or stock options	☒ **None**	
**12**	Receipt of equipment, materials, drugs, medical writing, gifts or other services	☐ **None**	
		Fujirebio in-kind reagents/equipment ADNI4 study	To institution
		Roche in-kind reagents/equipment ADNI4	To institution
**13**	Other financial or non-financial interests	☒ **None**	

Please place an "X" next to the following statement to indicate your agreement:

☒ I certify that I have answered every question and have not altered the wording of any of the questions on this form.

ICMJE DISCLOSURE FORM

**Date:**	5/8/2025
**Your Name:**	Susan M Landau
**Manuscript Title:**	Comparison of plasma biomarkers by race to detect Alzheimer's disease
**Manuscript Number (if known):**	ADJ-D-25-00365

In the interest of transparency, we ask you to disclose all relationships/activities/interests listed below that are related to the content of your manuscript. "Related" means any relation with for-profit or not-for-profit third parties whose interests may be affected by the content of the manuscript. Disclosure represents a commitment to transparency and does not necessarily indicate a bias. If you are in doubt about whether to list a relationship/activity/interest, it is preferable that you do so.

The author's relationships/activities/interests should be defined broadly. For example, if your manuscript pertains to the epidemiology of hypertension, you should declare all relationships with manufacturers of antihypertensive medication, even if that medication is not mentioned in the manuscript.

In item #1 below, report all support for the work reported in this manuscript without time limit. For all other items, the time frame for disclosure is the past 36 months.

		**Name all entities with whom you have this relationship or indicate none (add rows as needed)**	**Specifications/Comments (e.g., if payments were made to you or to your institution)**
**Time frame: Since the initial planning of the work**			
1	All support for the present manuscript (e.g., funding, provision of study materials, medical writing, article processing charges, etc.) **No time limit for this item.**	☐ **None**	
		U19AG24904	Institution
			Click the tab key to add additional rows.
**Time frame: past 36 months**			
2	Grants or contracts from any entity (if not indicated in item #1 above).	☒ **None**	
3	Royalties or licenses	☒ **None**	

		Name all entities with whom you have this relationship or indicate none (add rows as needed)	Specifications/Comments (e.g., if payments were made to you or to your institution)
**4**	Consulting fees	☐ **None**	
		Banner Health	Made to me
**5**	Payment or honoraria for lectures, presentations, speakers bureaus, manuscript writing or educational events	☐ **None**	
		Eisai	Speaking fees paid to me
		IMPACT-AD	Speaking fees paid to me
		J&J	Speaking fees paid to me
**6**	Payment for expert testimony	☒ **None**	
**7**	Support for attending meetings and/or travel	☐ **None**	
		ATRI	Made to me
		Shenzhen Bay Labs	Made to me
		J&J	Made to me
**8**	Patents planned, issued or pending	☒ **None**	
**9**	Participation on a Data Safety Monitoring Board or Advisory Board	☐ **None**	
		KeifeRx	Made to me
		NIH IPAT study	Made to me
**10**	Leadership or fiduciary role in other board, society, committee or advocacy group, paid or unpaid	☐ **None**	
		Editorial board, JAMA Neurology	unpaid

		Name all entities with whom you have this relationship or indicate none (add rows as needed)	Specifications/Comments (e.g., if payments were made to you or to your institution)
**11**	Stock or stock options	☒ **None**	
**12**	Receipt of equipment, materials, drugs, medical writing, gifts or other services	☒ **None**	
**13**	Other financial or non-financial interests	☐ **None**	
		Research support on the US POINTER neuroimaging ancillary study from the Alzhiemer's Association	

Please place an "X" next to the following statement to indicate your agreement:

☒ I certify that I have answered every question and have not altered the wording of any of the questions on this form.

Date: 5/9/2025

Your Name: Monica Rivera Mindt

Manuscript Title: Comparison of plasma biomarkers by race to detect Alzheimer's disease

Manuscript Number (if known): ADJ-D-25-00365

In the interest of transparency, we ask you to disclose all relationships/activities/interests listed below that are related to the content of your manuscript. "Related" means any relation with for-profit or not-for-profit third parties whose interests may be affected by the content of the manuscript. Disclosure represents a commitment to transparency and does not necessarily indicate a bias. If you are in doubt about whether to list a relationship/activity/interest, it is preferable that you do so.

The author's relationships/activities/interests should be defined broadly. For example, if your manuscript pertains to the epidemiology of hypertension, you should declare all relationships with manufacturers of antihypertensive medication, even if that medication is not mentioned in the manuscript.

In item #1 below, report all support for the work reported in this manuscript without time limit. For all other items, the time frame for disclosure is the past 36 months.

		Name all entities with whom you have this relationship or indicate none (add rows as needed)	Specifications/Comments (e.g., if payments were made to you or to your institution)
		**Time frame: Since the initial planning of the work**	
1	All support for the present manuscript (e.g., funding, provision of study materials, medical writing, article processing charges, etc.) **No time limit for this item.**	☐ **None**	
		NIH	
			Click the tab key to add additional rows.
		**Time frame: past 36 months**	
2	Grants or contracts from any entity (if not indicated in item #1 above).	☐ **None**	
		NIH-funded grants: U19AG024904; U19AG078109-01; R01AG066471; R56AG075744; R13AG071313-01; & R01AG065110 - 01A1; SC3GM141996	
3	Royalties or licenses	☒ **None**	

		Name all entities with whom you have this relationship or indicate none (add rows as needed)	Specifications/Comments (e.g., if payments were made to you or to your institution)
4	Consulting fees	☒ **None**	
5	Payment or honoraria for lectures, presentations, speakers bureaus, manuscript writing or educational events	☐ **None**	
		University of Texas Rio Grand Valley	
		Clinical Trials on Alzheimer's Disease 2023 Conference	
		American Neurological Association Conference	
		Catamount Medical Education Presentation	
		SNS Annual Symposium 2025	
6	Payment for expert testimony	☒ **None**	
7	Support for attending meetings and/or travel	☐ **None**	
		NIH	
8	Patents planned, issued or pending	☒ **None**	
9	Participation on a Data Safety Monitoring Board or Advisory Board	☐ **None**	
		NCRAD Executive Committee	ALL-FTD External Advisory Board
		Brown University Center for Alzheimer's Disease Research	
		DSMB: Natives Engaged in Alzheimer's Research (NEAR) (NIH-funded grant) Advisory Board Member: National Centralized Repository for ADRD (NCRAD) Executive Committee; University of Texas Rio Grand Valley Resource Center for Minority Aging Research Advisory Board; University of Washington Alzheimer's Disease Research Center (ADRC) Advisory Board; UCSF Alzheimer's Disease Research Center (ADRC) Advisory Board; Mayo Clinic Alzheimer's Disease Research Center (ADRC) Advisory Board; ALL-FTD External Advisory Board; Brown University Center for Alzheimer's Disease Research; Centers	

		Name all entities with whom you have this relationship or indicate none (add rows as needed)	Specifications/Comments (e.g., if payments were made to you or to your institution)
		for Disease Control and Prevention (CDC) BOLD Public Health Center of Excellence on Dementia Risk Reduction Expert Panel; Alzheimer's Association NYC Chapter Board of Directors	
**10**	Leadership or fiduciary role in other board, society, committee or advocacy group, paid or unpaid	☐ **None**	
		Harlem Community and Academic Partnership	
		Alzheimer's Association – NYC Board	
**11**	Stock or stock options	☒ **None**	
**12**	Receipt of equipment, materials, drugs, medical writing, gifts or other services	☒ **None**	
**13**	Other financial or non-financial interests	☒ **None**	

Please place an "X" next to the following statement to indicate your agreement:

☒ I certify that I have answered every question and have not altered the wording of any of the questions on this form.

ICMJE DISCLOSURE FORM

**Date:**	5/9/2025
**Your Name:**	Michael W. Weiner, MD
**Manuscript Title:**	Comparison of plasma biomarkers by race to detect Alzheimer's disease
**Manuscript Number (if known):**	ADJ-D-25-00365

In the interest of transparency, we ask you to disclose all relationships/activities/interests listed below that are related to the content of your manuscript. "Related" means any relation with for-profit or not-for-profit third parties whose interests may be affected by the content of the manuscript. Disclosure represents a commitment to transparency and does not necessarily indicate a bias. If you are in doubt about whether to list a relationship/activity/interest, it is preferable that you do so.

The author's relationships/activities/interests should be defined broadly. For example, if your manuscript pertains to the epidemiology of hypertension, you should declare all relationships with manufacturers of antihypertensive medication, even if that medication is not mentioned in the manuscript.

In item #1 below, report all support for the work reported in this manuscript without time limit. For all other items, the time frame for disclosure is the past 36 months.

		**Name all entities with whom you have this relationship or indicate none (add rows as needed)**	**Specifications/Comments (e.g., if payments were made to you or to your institution)**
**Time frame: Since the initial planning of the work**			
**1**	All support for the present manuscript (e.g., funding, provision of study materials, medical writing, article processing charges, etc.) **No time limit for this item.**	☐ **None**	
		NIH Grant: 2 U19 AG024904.16	Payments were made to my institution.
**Time frame: past 36 months**			
**2**	Grants or contracts from any entity (if not indicated in item #1 above).	☐ **None**	
		NIH Grant: 5U2CAG060426-04	Payments were made to my institution.
		NIH Grant: 5R01AG058676-02	Payments were made to my institution.
		NIH Grant: 1RF1AG059009-01	Payments were made to my institution.
		NIH Grant: R33 AG062867	Payments were made to my institution.
		NIH Grant: 1R01NS119651-01	Payments were made to my institution.
		NIH Grant: RF1AG062196	Payments were made to my institution.
		NIH Grant: R56AG075744-01A1	Payments were made to my institution.
		Additional support from Department of Defense (DOD)	Payments were made to my institution.
		Additional support from: California Department of Public Health (CDPH)	Payments were made to my institution.

		Name all entities with whom you have this relationship or indicate none (add rows as needed)	Specifications/Comments (e.g., if payments were made to you or to your institution)
		Additional support from: Siemens	Payments were made to my institution.
		Additional support from: Biogen	Payments were made to my institution.
		Additional support from: Hillblom Foundation	Payments were made to my institution.
		Additional support from: Alzheimer's Association	Payments were made to my institution.
		Additional support from: Johnson & Johnson	Payments were made to my institution.
		Additional support from: Kevin and Connie Shanahan	Payments were made to my institution.
		Additional support from: GE HealthCare	Payments were made to my institution.
		Additional support from: VUmc	Payments were made to my institution.
		Additional support from: Australian Catholic University (HBI-BHR)	Payments were made to my institution.
		Additional support from: The Stroke Foundation	Payments were made to my institution.
		Additional support from: Veterans Administration	Payments were made to my institution.
**3**	Royalties or licenses	X **None**	
**4**	Consulting fees	☐ **None**	
		Acadia Pharmaceuticals	Payment was made directly to me.
		Boxer Capital	Payment was made directly to me.
		Cerecin/BioClinica	Payment was made directly to me.
		Dementia Society of Japan	Payment was made directly to me.
		Eisai	Payment was made directly to me.
		Guidepoint	Payment was made directly to me.
		Health & Wellness Partners	Payment was made directly to me.
		Indiana U.	Payment was made directly to me.
		LCN Consulting	Payment was made directly to me.
		Merck, Sharp & Dohme	Payment was made directly to me.
		Duke U.	Payment was made directly to me.
		Owkin France	Payment was made directly to me.
		NovoNordisk	Payment was made directly to me.
		ProMIS Neurosciences	Payment was made directly to me.
		Prova Education	Payment was made directly to me.
		Sai MedPartners	Payment was made directly to me.
		T3D Therapeutics	Payment was made directly to me.
		U. Southern CA	Payment was made directly to me.
		WebMD	Payment was made directly to me.
		MEDA Corp.	Payment was made directly to me.
**5**	Payment or honoraria for lectures, presentations, speakers	☐ **None**	
		Merck, Sharp & Dohme	Payment was made directly to me.

		Name all entities with whom you have this relationship or indicate none (add rows as needed)	Specifications/Comments (e.g., if payments were made to you or to your institution)
	bureaus, manuscript writing or educational events	BrightFocus Foundation	Payment was made directly to me.
		China Assoc. for AD	Payment was made directly to me.
		Taipei Medical U.	Payment was made directly to me.
		Cleveland Clinic	Payment was made directly to me.
		Banner Health	Payment was made directly to me.
		AD/PD Congress	Payment was made directly to me.
		Foundation of Learning Health Society, Japan	Payment was made directly to me.
		Japan Society for Dementia Research	Payment was made directly to me.
		U. Toulouse	Payment was made directly to me.
		Korean Dementia Society	Payment was made directly to me.
		U. Madison Wisconsin	Payment was made directly to me.
**6**	Payment for expert testimony	X **None**	
**7**	Support for attending meetings and/or travel	□ **None**	
		Johnson & Johnson	Payment was made directly to me or to airlines/hotels/transpo.
		Merck, Sharp & Dohme	Payment was made directly to me or to airlines/hotels/transpo.
		U. Southern CA	Payment was made directly to me or to airlines/hotels/transpo.
		Cleveland Clinic	Payment was made directly to me or to airlines/hotels/transpo.
		Banner Health	Payment was made directly to me or to airlines/hotels/transpo.
		AD/PD Congress	Payment was made directly to me or to airlines/hotels/transpo.
		Foundation of Learning Health Society, Japan	Payment was made directly to me or to airlines/hotels/transpo.
		Japan Society for Dementia Research	Payment was made directly to me or to airlines/hotels/transpo.
		U. Toulouse	Payment was made directly to me or to airlines/hotels/transpo.
		Korean Dementia Society	Payment was made directly to me or to airlines/hotels/transpo.
		Kenes	Payment was made directly to me or to airlines/hotels/transpo.
		CTAD Congress	Payment was made directly to me or to airlines/hotels/transpo.
		National Center for Geriatrics and Gerontology, Japan	Payment was made directly to me or to airlines/hotels/transpo.

		Name all entities with whom you have this relationship or indicate none (add rows as needed)	Specifications/Comments (e.g., if payments were made to you or to your institution)
**8**	Patents planned, issued or pending	X **None**	
**9**	Participation on a Data Safety Monitoring Board or Advisory Board	X **None**	
**10**	Leadership or fiduciary role in other board, society, committee or advocacy group, paid or unpaid	X **None**	
**11**	Stock or stock options	☐ **None**	
		Alzeca	Stock options held.
		ALZpath	Stock options held.
		Alzheon	Stock options held.
		Anven	Stock options held.
**12**	Receipt of equipment, materials, drugs, medical writing, gifts or other services	X **None**	
**13**	Other financial or non-financial interests	X **None**	

Please place an "X" next to the following statement to indicate your agreement:

X I certify that I have answered every question and have not altered the wording of any of the questions on this form.
