# Supplementary material for: Comparison of plasma p‐tau217/Aβ42, p‐tau217, and Aβ42/Aβ40 biomarkers by race to detect Alzheimer's disease
Source: Alzheimers Dement. 2025 Aug 13;21(8):e70469. doi: 10.1002/alz.70469 (PMC12344574; doi:10.1002/alz.70469)
Supplement: Supplementary file 2 — Supporting Information [file ALZ-21-e70469-s001.docx]

# 5. Supplementary Material

# Comparison of plasma p-tau_217_/Aβ_42_, p-tau_217_, and Aβ_42_/Aβ_40_ biomarkers by race to detect Alzheimer’s disease

Katheryn A.Q. Cousins, PhD, Magdalena Korecka, PhD, Yang Wan, MS, Amberley Vulaj, MA, Christopher Brown, MD, PhD, Thomas F. Tropea, DO, Edward B. Lee, MD, PhD, Duygu Tosun, PhD, Susan M. Landau, PhD, Ozioma Okonkwo, PhD, Monica Rivera Mindt, PhD, Michael W. Weiner, MD, David J. Irwin, MD, David A. Wolk, MD, Leslie M. Shaw, PhD, and the Alzheimer’s Disease Neuroimaging Initiative

## 5.1 Plasma Assay Methods

| **p-tau217/Aβ42** | **p-tau217** | **Aβ42/A40** | **Aβ42** | **Aβ40** |
| --- | --- | --- | --- | --- |
| Y=1.02X+0.0001 | Y=0.98X+0.003 | Y=0.90X+009 | Y=0.87X+3.2 | Y=0.92X+22.5 |
| r2=0.990 | r2=0.992 | r2=0.800 | r2=0.770 | r2=0.790 |

##### **eTable 1: Test/re-test performance for plasma AD biomarkers in the ADNI study.** A total of 201 plasma samples from the ADNI4 study, not analyzed as part of the current study, were analysed in 6 batches. In the linear regression equations above Y values were the “test” and X values were the “re-test” samples. Re-testing was performed 1 to 2 days following “test” analyses.

## 5.2 Part 1: All UPenn participants

### 5.2.1 ROC analyses by Cognitive Status

Fisher’s tests determined if classification accuracy (correct vs. errors) differed by cognitive status. Lower accuracy in cognitively normal compared to MCI/Dementia was not significant for plasma p-tau_217_/Aβ_42_ (OR=0.24, 95%CI=0.03 – 1.09, *p*=0.051), p-tau_217_ (OR=0.24, 95%CI=0.03 – 1.14, *p*=0.053), or Aβ_42_/Aβ_40_ (OR=2.04, 95%CI=0.57 – 7.37, *p*=0.24).


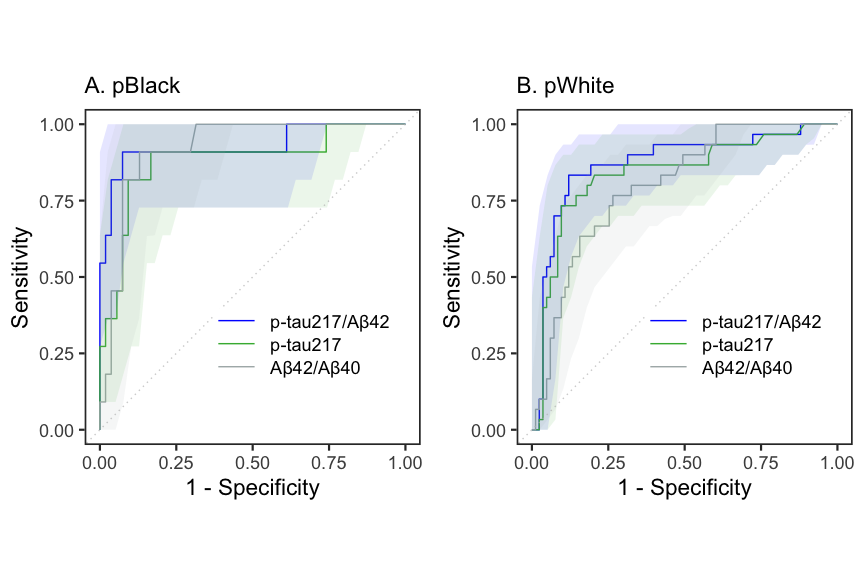


##### **eFigure 1. Receiver Operating Characteristic (ROC) analyses in UPenn cognitively normal participants to discriminate Amyloid+ from Amyloid-** (n=178). ROC curves plot sensitivity vs. specificity. Color indicates biomarker: p-tau_217_/Aβ_42_ (blue), p-tau_217_ (green), Aβ_42_/Aβ_40_ (grey). Panels are separated by race: (**A.**) pBlack, (**B.**) pWhite.


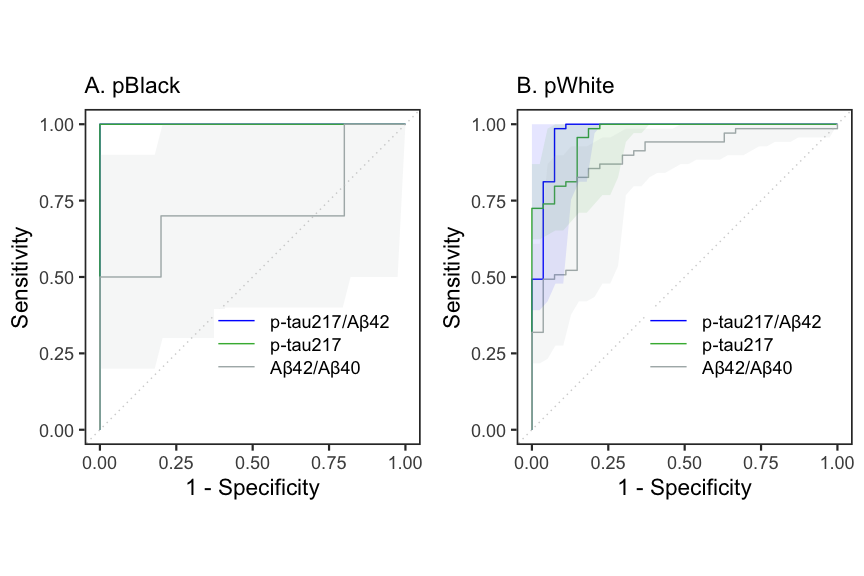


##### **eFigure 2. Receiver Operating Characteristic (ROC) analyses in UPenn participants with MCI/Dementia to discriminate Amyloid+ from Amyloid-** (n=111). ROC curves plot sensitivity vs. specificity. Color indicates biomarker: p-tau_217_/Aβ_42_ (blue), p-tau_217_ (green), Aβ_42_/Aβ_40_ (grey). Panels are separated by race: (**A.**) pBlack, (**B.**) pWhite. **NOTE: For pBlack, p-tau_217_/Aβ_42_ and p-tau_217_ have the same ROC curve (AUC=1), thus p-tau_217_/Aβ_42_ curve (solid blue) cannot be seen (see Figure 2)**.

### 5.2.2 Comparisons by Race


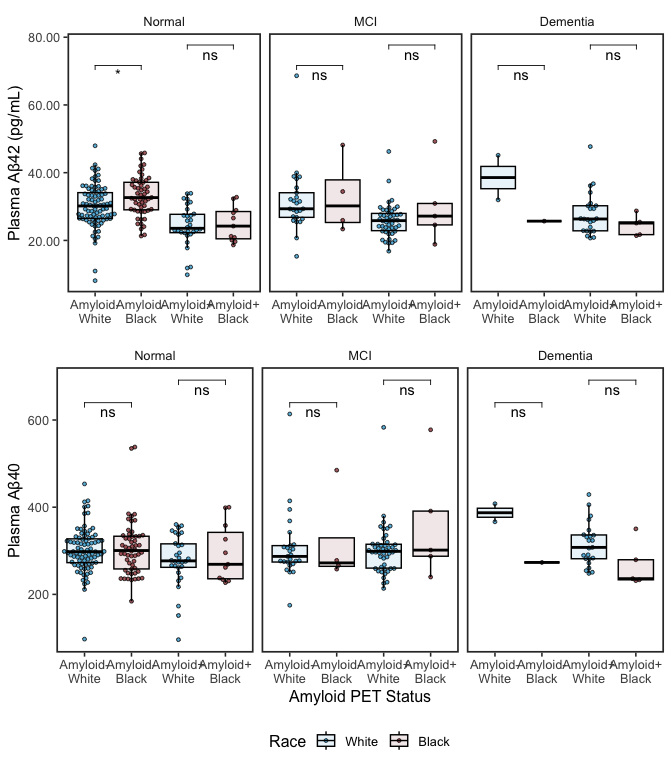


##### **eFigure 3. Plasma Aβ analytes by race (White, Black), Amyloid status (+/-) in all UPenn participants** (n=289). Boxplots show median, interquartile range (IQR), and outliers for each plasma biomarker. Color indicates self-identified White (blue) and Black/African American (red) participants. Solid horizontal line indicates 0.95 specificity threshold; broken horizonal lines indicate 0.95 sensitivity threshold (Table 1). Asterisks represent nominal Bonferroni adjusted *p*-values from Wilcoxon pairwise comparisons (* *p*<0.05 or not significant [ns]).

| **A. UPenn Training Sample (all)** | n | Plasma Aβ42 | Plasma Aβ40 | Plasma p-tau217 | Plasma Aβ42/Aβ40 | Plasma ptau217/Aβ42 |
| --- | --- | --- | --- | --- | --- | --- |
| White Normal Amyloid- | 83 | 30.122 (6.344) | 301.880 (50.937) | 0.163 (0.445) | 0.100 (0.014) | 0.006 (0.018) |
| Black Normal Amyloid- | 54 | 33.138 (5.912) | 306.152 (64.626) | 0.108 (0.083) | 0.110 (0.014) | 0.003 (0.002) |
| White MCI Amyloid- | 25 | 31.652 (9.595) | 307.491 (80.115) | 0.119 (0.076) | 0.102 (0.010) | 0.004 (0.003) |
| Black MCI Amyloid- | 4 | 32.980 (11.177) | 321.810 (109.035) | 0.097 (0.047) | 0.103 (0.015) | 0.003 (0.002) |
| White Dementia Amyloid- | 2 | 38.560 (9.306) | 387.275 (29.239) | 0.253 (0.110) | 0.099 (0.017) | 0.007 (0.005) |
| Black Dementia Amyloid- | 1 | 25.690 (NA) | 273.260 (NA) | 0.040 (NA) | 0.094 (NA) | 0.002 (NA) |
| White Normal Amyloid+ | 30 | 24.337 (6.114) | 277.393 (61.211) | 0.277 (0.222) | 0.088 (0.010) | 0.013 (0.012) |
| Black Normal Amyloid+ | 11 | 24.859 (5.160) | 294.453 (66.504) | 0.290 (0.139) | 0.085 (0.010) | 0.012 (0.005) |
| White MCI Amyloid+ | 46 | 25.894 (5.002) | 300.747 (57.286) | 0.616 (0.561) | 0.086 (0.009) | 0.023 (0.016) |
| Black MCI Amyloid+ | 5 | 30.152 (11.538) | 359.514 (133.625) | 0.700 (0.271) | 0.085 (0.016) | 0.025 (0.012) |
| White Dementia Amyloid+ | 23 | 27.798 (6.356) | 315.007 (49.103) | 0.877 (0.543) | 0.089 (0.021) | 0.033 (0.022) |
| Black Dementia Amyloid+ | 5 | 24.474 (3.015) | 266.178 (51.077) | 0.685 (0.167) | 0.094 (0.014) | 0.028 (0.009) |

##### **eTable 2a: Mean and standard deviation for plasma biomarkers in UPenn Sample.** Mean (standard deviation) are reported for all plasma biomarkers for each group. “NA” indicates SD could not be computed from n=1.

| **B. ADNI Test Sample (all)** | n | Plasma Aβ42 | Plasma Aβ40 | Plasma p-tau217 | Plasma Aβ42/Aβ40 | Plasma ptau217/Aβ42 |
| --- | --- | --- | --- | --- | --- | --- |
| White Normal Amyloid- | 256 | 27.439 (6.482) | 296.357 (72.071) | 0.140 (0.173) | 0.096 (0.039) | 0.006 (0.008) |
| Black Normal Amyloid- | 54 | 26.196 (4.691) | 280.066 (45.492) | 0.101 (0.096) | 0.094 (0.012) | 0.004 (0.004) |
| White MCI Amyloid- | 138 | 28.512 (5.940) | 315.289 (69.847) | 0.159 (0.173) | 0.091 (0.011) | 0.007 (0.013) |
| Black MCI Amyloid- | 15 | 27.969 (6.264) | 288.841 (46.811) | 0.083 (0.036) | 0.096 (0.013) | 0.003 (0.002) |
| White Normal Amyloid+ | 137 | 23.795 (5.302) | 296.261 (64.436) | 0.302 (0.202) | 0.081 (0.011) | 0.014 (0.013) |
| Black Normal Amyloid+ | 16 | 25.234 (5.283) | 290.098 (50.038) | 0.150 (0.083) | 0.087 (0.010) | 0.006 (0.003) |
| White MCI Amyloid+ | 210 | 28.861 (67.691) | 331.775 (339.557) | 0.584 (0.368) | 0.079 (0.012) | 0.025 (0.017) |
| Black MCI Amyloid+ | 20 | 22.674 (5.413) | 280.840 (63.253) | 0.618 (0.396) | 0.081 (0.007) | 0.027 (0.017) |

##### **eTable 2b: Mean and standard deviation for plasma biomarkers in ADNI Sample.** Mean (standard deviation) are reported for all plasma biomarkers for each group.

| **Plasma ptau217/Aβ42** | **β** | **95%CI** | **p** | | **Adj p** | |  |
| --- | --- | --- | --- | --- | --- | --- | --- |
| PET Status = Amyloid+ | 1.353 | 1.129 – 1.576 | <0.0001 | | <0.0001 | |  |
| Race = Black | 0.126 | -0.121 – 0.372 | 0.3164 | | 0.9492 | |  |
| Age at Plasma (years) | 0.007 | -0.007 – 0.021 | 0.3290 | | 0.9869 | |  |
| Sex = Male | 0.085 | -0.098 – 0.268 | 0.3606 | | 1.0000 | |  |
| ADI = Medium Deprivation | -0.160 | -0.353 – 0.033 | 0.1032 | | 0.3096 | |  |
| ADI = High Deprivation | -0.156 | -0.481 – 0.169 | 0.3448 | | 1.0000 | |  |
| Cognition = MCI | 0.310 | 0.094 – 0.526 | 0.0052 | | 0.0155 | |  |
| Cognition = Dementia | 0.738 | 0.43 – 1.047 | <0.0001 | | <0.0001 | |  |
| APOE≥1 ε4 allele(s) | 0.051 | -0.142 – 0.243 | 0.6059 | | 1.0000 | |  |
| History of Diabetes = Yes | -0.067 | -0.308 – 0.174 | 0.5859 | | 1.0000 | |  |
| Body Mass Index (BMI) | -0.010 | -0.029 – 0.008 | 0.2832 | | 0.8497 | |  |
| **Plasma p-tau217** | **β** | **95%CI** | **p** | | **Adj p** | |  |
| PET Status = Amyloid+ | 1.180 | 0.96 – 1.4 | <0.0001 | | <0.0001 | |  |
| Race = Black | 0.170 | -0.073 – 0.412 | 0.1687 | | 0.5062 | |  |
| Age at Plasma (years) | 0.011 | -0.003 – 0.025 | 0.1120 | | 0.3360 | |  |
| Sex = Male | 0.028 | -0.152 – 0.208 | 0.7605 | | 1.0000 | |  |
| ADI = Medium Deprivation | -0.190 | -0.38 – 0 | 0.0500 | | 0.1501 | |  |
| ADI = High Deprivation | -0.194 | -0.513 – 0.126 | 0.2337 | | 0.7010 | |  |
| Cognition = MCI | 0.368 | 0.155 – 0.581 | 0.0008 | | 0.0023 | |  |
| Cognition = Dementia | 0.862 | 0.559 – 1.166 | <0.0001 | | <0.0001 | |  |
| APOE≥1 ε4 allele(s) | -0.025 | -0.215 – 0.165 | 0.7960 | | 1.0000 | |  |
| History of Diabetes = Yes | -0.067 | -0.304 – 0.17 | 0.5797 | | 1.0000 | |  |
| Body Mass Index (BMI) | -0.003 | -0.021 – 0.015 | 0.7632 | | 1.0000 | |  |
| **Plasma Aβ42/40** | **β** | **95%CI** | | **p** | | **Adj p** | |
| PET Status = Amyloid+ | -0.123 | -0.172 – -0.075 | | <0.0001 | | <0.0001 | |
| Race = Black | 0.075 | 0.021 – 0.128 | | 0.0061 | | 0.0184 | |
| Age at Plasma (years) | -0.004 | -0.007 – -0.001 | | 0.0106 | | 0.0318 | |
| Sex = Male | 0.014 | -0.026 – 0.053 | | 0.4895 | | 1.0000 | |
| ADI = Medium Deprivation | -0.021 | -0.062 – 0.021 | | 0.3247 | | 0.9742 | |
| ADI = High Deprivation | 0.022 | -0.048 – 0.092 | | 0.5302 | | 1.0000 | |
| Cognition = MCI | 0.013 | -0.034 – 0.059 | | 0.5953 | | 1.0000 | |
| Cognition = Dementia | 0.027 | -0.039 – 0.094 | | 0.4164 | | 1.0000 | |
| APOE≥1 ε4 allele(s) | -0.062 | -0.104 – -0.021 | | 0.0035 | | 0.0104 | |
| History of Diabetes = Yes | -0.097 | -0.149 – -0.045 | | 0.0003 | | 0.0008 | |
| Body Mass Index (BMI) | 0.000 | -0.004 – 0.004 | | 0.9657 | | 1.0000 | |

##### **eTable 3A. Linear Models in all UPenn participants with BMI**. Linear models tested plasma biomarkers (log-transformed) as a function of Amyloid status (Amyloid-, Amyloid+) and self-identified race (White, Black), including age, sex, area deprivation index (ADI), cognition (normal, impaired), APOE ε4, and history of diabetes as covariates. Nominal and Bonferroni adjusted (Adj) *p*-values are reported.

| **Plasma Aβ42** | **β** | **95%CI** | **p** |
| --- | --- | --- | --- |
| PET Status = Amyloid+ | -0.207 | -0.276 – -0.137 | <0.0001 |
| Race = Black | 0.072 | -0.003 – 0.147 | 0.0582 |
| Age at Plasma (years) | 0.005 | 0 – 0.009 | 0.0344 |
| Sex = Male | -0.050 | -0.109 – 0.01 | 0.1028 |
| ADI = Medium Deprivation | -0.026 | -0.088 – 0.036 | 0.4124 |
| ADI = High Deprivation | -0.033 | -0.131 – 0.066 | 0.5137 |
| Cognition = MCI | 0.056 | -0.013 – 0.126 | 0.1127 |
| Cognition = Dementia | 0.123 | 0.022 – 0.224 | 0.0168 |
| APOE≥1 ε4 allele(s) | -0.066 | -0.127 – -0.005 | 0.0348 |
| History of Diabetes = Yes | 0.026 | -0.048 – 0.1 | 0.4917 |
| **Plasma Aβ40** | **β** | **95%CI** | **p** |
| PET Status = Amyloid+ | -0.072 | -0.128 – -0.016 | 0.0122 |
| Race = Black | -0.013 | -0.073 – 0.047 | 0.6648 |
| Age at Plasma (years) | 0.009 | 0.005 – 0.013 | <0.0001 |
| Sex = Male | -0.061 | -0.109 – -0.013 | 0.0135 |
| ADI = Medium Deprivation | -0.003 | -0.053 – 0.047 | 0.9046 |
| ADI = High Deprivation | -0.047 | -0.127 – 0.033 | 0.2456 |
| Cognition = MCI | 0.042 | -0.014 – 0.098 | 0.1448 |
| Cognition = Dementia | 0.091 | 0.01 – 0.173 | 0.0278 |
| APOE≥1 ε4 allele(s) | -0.007 | -0.056 – 0.043 | 0.7921 |
| History of Diabetes = Yes | 0.118 | 0.058 – 0.178 | 0.0001 |

##### **eTable 3B. Linear Models in all UPenn participants: Aβ42 and Aβ40 analytes**. Linear models tested plasma biomarkers (log-transformed) as a function of Amyloid status (Amyloid-, Amyloid+) and self-identified race (White, Black), including age, sex, area deprivation index (ADI), cognition (normal, impaired), APOE ε4, and history of diabetes as covariates.

| **Plasma ptau217/Aβ42** | **β** | **95%CI** | **p** | | **Adj p** | |  |
| --- | --- | --- | --- | --- | --- | --- | --- |
| PET Status = Amyloid+ | 1.353 | 1.129 – 1.576 | <0.0001 | | <0.0001 | |  |
| Race = Black | 0.126 | -0.121 – 0.372 | 0.3164 | | 0.9492 | |  |
| Age at Plasma (years) | 0.007 | -0.007 – 0.021 | 0.3290 | | 0.9869 | |  |
| Sex = Male | 0.085 | -0.098 – 0.268 | 0.3606 | | 1.0000 | |  |
| ADI = Medium Deprivation | -0.160 | -0.353 – 0.033 | 0.1032 | | 0.3096 | |  |
| ADI = High Deprivation | -0.156 | -0.481 – 0.169 | 0.3448 | | 1.0000 | |  |
| Cognition = MCI | 0.310 | 0.094 – 0.526 | 0.0052 | | 0.0155 | |  |
| Cognition = Dementia | 0.738 | 0.43 – 1.047 | <0.0001 | | <0.0001 | |  |
| APOE≥1 ε4 allele(s) | 0.051 | -0.142 – 0.243 | 0.6059 | | 1.0000 | |  |
| History of Diabetes = Yes | -0.067 | -0.308 – 0.174 | 0.5859 | | 1.0000 | |  |
| Body Mass Index (BMI) | -0.010 | -0.029 – 0.008 | 0.2832 | | 0.8497 | |  |
| **Plasma p-tau217** | **β** | **95%CI** | **p** | | **Adj p** | |  |
| PET Status = Amyloid+ | 1.180 | 0.96 – 1.4 | <0.0001 | | <0.0001 | |  |
| Race = Black | 0.170 | -0.073 – 0.412 | 0.1687 | | 0.5062 | |  |
| Age at Plasma (years) | 0.011 | -0.003 – 0.025 | 0.1120 | | 0.3360 | |  |
| Sex = Male | 0.028 | -0.152 – 0.208 | 0.7605 | | 1.0000 | |  |
| ADI = Medium Deprivation | -0.190 | -0.38 – 0 | 0.0500 | | 0.1501 | |  |
| ADI = High Deprivation | -0.194 | -0.513 – 0.126 | 0.2337 | | 0.7010 | |  |
| Cognition = MCI | 0.368 | 0.155 – 0.581 | 0.0008 | | 0.0023 | |  |
| Cognition = Dementia | 0.862 | 0.559 – 1.166 | <0.0001 | | <0.0001 | |  |
| APOE≥1 ε4 allele(s) | -0.025 | -0.215 – 0.165 | 0.7960 | | 1.0000 | |  |
| History of Diabetes = Yes | -0.067 | -0.304 – 0.17 | 0.5797 | | 1.0000 | |  |
| Body Mass Index (BMI) | -0.003 | -0.021 – 0.015 | 0.7632 | | 1.0000 | |  |
| **Plasma Aβ42/40** | **β** | **95%CI** | | **p** | | **Adj p** | |
| PET Status = Amyloid+ | -0.123 | -0.172 – -0.075 | | <0.0001 | | <0.0001 | |
| Race = Black | 0.075 | 0.021 – 0.128 | | 0.0061 | | 0.0184 | |
| Age at Plasma (years) | -0.004 | -0.007 – -0.001 | | 0.0106 | | 0.0318 | |
| Sex = Male | 0.014 | -0.026 – 0.053 | | 0.4895 | | 1.0000 | |
| ADI = Medium Deprivation | -0.021 | -0.062 – 0.021 | | 0.3247 | | 0.9742 | |
| ADI = High Deprivation | 0.022 | -0.048 – 0.092 | | 0.5302 | | 1.0000 | |
| Cognition = MCI | 0.013 | -0.034 – 0.059 | | 0.5953 | | 1.0000 | |
| Cognition = Dementia | 0.027 | -0.039 – 0.094 | | 0.4164 | | 1.0000 | |
| APOE≥1 ε4 allele(s) | -0.062 | -0.104 – -0.021 | | 0.0035 | | 0.0104 | |
| History of Diabetes = Yes | -0.097 | -0.149 – -0.045 | | 0.0003 | | 0.0008 | |
| Body Mass Index (BMI) | 0.000 | -0.004 – 0.004 | | 0.9657 | | 1.0000 | |

##### **eTable 3C. Linear Models in all UPenn participants with BMI as a covariate**. Linear models tested plasma biomarkers (log-transformed) as a function of Amyloid status (Amyloid-, Amyloid+) and self-identified race (White, Black), including age, sex, area deprivation index (ADI), cognition (normal, impaired), APOE ε4, history of diabetes, and BMI as covariates. Nominal and Bonferroni adjusted (Adj) *p*-values are reported.

## 5.3 Matched UPenn Sample: Comparisons by Race

| **Plasma ptau217/Aβ42** | **β** | **95%CI** | **p** | | | **Adj p** | |
| --- | --- | --- | --- | --- | --- | --- | --- |
| PET Status = Amyloid+ | 1.137 | 0.844 – 1.429 | <0.0001 | | | <0.0001 | |
| Race = Black | 0.007 | -0.243 – 0.258 | 0.9546 | | | 1.0000 | |
| Age at Plasma (years) | 0.028 | 0.008 – 0.049 | 0.0074 | | | 0.0223 | |
| Sex = Male | 0.192 | -0.156 – 0.541 | 0.2774 | | | 0.8322 | |
| Cognition = MCI | 0.248 | -0.12 – 0.615 | 0.1850 | | | 0.5550 | |
| Cognition = Dementia | 0.881 | 0.438 – 1.324 | 0.0001 | | | 0.0004 | |
| ADI = Medium Deprivation | -0.181 | -0.432 – 0.07 | 0.1556 | | | 0.4668 | |
| ADI = High Deprivation | -0.089 | -0.419 – 0.242 | 0.5967 | | | 1.0000 | |
| APOE≥1 ε4 allele(s) | 0.135 | -0.111 – 0.381 | 0.2790 | | | 0.8369 | |
| History of Diabetes = Yes | 0.002 | -0.256 – 0.26 | 0.9867 | | | 1.0000 | |
| **Plasma p-tau217** | **β** | **95%CI** | **p** | | | **Adj p** | |
| PET Status = Amyloid+ | 0.934 | 0.641 – 1.228 | <0.0001 | | | <0.0001 | |
| Race = Black | 0.115 | -0.136 – 0.366 | 0.3671 | | | 1.0000 | |
| Age at Plasma (years) | 0.033 | 0.012 – 0.053 | 0.0023 | | | 0.0070 | |
| Sex = Male | 0.086 | -0.264 – 0.436 | 0.6273 | | | 1.0000 | |
| Cognition = MCI | 0.315 | -0.053 – 0.684 | 0.0930 | | | 0.2790 | |
| Cognition = Dementia | 0.941 | 0.496 – 1.385 | 0.0001 | | | 0.0002 | |
| ADI = Medium Deprivation | -0.226 | -0.478 – 0.026 | 0.0779 | | | 0.2336 | |
| ADI = High Deprivation | -0.146 | -0.478 – 0.186 | 0.3861 | | | 1.0000 | |
| APOE≥1 ε4 allele(s) | 0.041 | -0.206 – 0.288 | 0.7445 | | | 1.0000 | |
| History of Diabetes = Yes | 0.049 | -0.21 – 0.307 | 0.7107 | | | 1.0000 | |
| **Plasma Aβ42/40** | **β** | **95%CI** | | **p** | **Adj p** | |  |
| PET Status = Amyloid+ | -0.121 | -0.192 – -0.05 | | 0.0009 | 0.0028 | |  |
| Race = Black | 0.108 | 0.047 – 0.168 | | 0.0006 | 0.0018 | |  |
| Age at Plasma (years) | -0.008 | -0.013 – -0.003 | | 0.0032 | 0.0097 | |  |
| Sex = Male | -0.028 | -0.112 – 0.057 | | 0.5144 | 1.0000 | |  |
| Cognition = MCI | 0.043 | -0.045 – 0.132 | | 0.3360 | 1.0000 | |  |
| Cognition = Dementia | 0.029 | -0.078 – 0.136 | | 0.5946 | 1.0000 | |  |
| ADI = Medium Deprivation | -0.039 | -0.1 – 0.022 | | 0.2066 | 0.6199 | |  |
| ADI = High Deprivation | -0.010 | -0.09 – 0.07 | | 0.8092 | 1.0000 | |  |
| APOE≥1 ε4 allele(s) | -0.083 | -0.142 – -0.023 | | 0.0067 | 0.0200 | |  |
| History of Diabetes = Yes | -0.109 | -0.171 – -0.047 | | 0.0007 | 0.0022 | |  |

##### **eTable 4. Plasma biomarkers by Amyloid status and race in Matched UPenn sample**. Linear models tested plasma biomarkers (log-transformed) as a function of Amyloid status (Amyloid-, Amyloid+) and self-identified race (White, Black), including age, sex, area deprivation index (ADI), cognition (normal, impaired), and APOE ε4 as covariates. Nominal and Bonferroni adjusted (Adj) *p*-values are reported.

| **Plasma ptau217/Aβ42** | **β** | **95%CI** | **p** | | | **Adj p** | |
| --- | --- | --- | --- | --- | --- | --- | --- |
| Race = Black | -0.585 | -1.626 – 0.455 | 0.2680 | | | 0.8040 | |
| Aβ PET SUVR | 2.349 | 1.587 – 3.111 | <0.0001 | | | <0.0001 | |
| Age at Plasma (years) | 0.029 | 0.009 – 0.048 | 0.0044 | | | 0.0132 | |
| Plasma to AβPET | 0.192 | 0.03 – 0.353 | 0.0205 | | | 0.0614 | |
| ADI = Medium Deprivation | -0.138 | -0.375 – 0.099 | 0.2512 | | | 0.7535 | |
| ADI = High Deprivation | 0.030 | -0.286 – 0.346 | 0.8521 | | | 1.0000 | |
| Cognition = MCI | 0.279 | -0.063 – 0.621 | 0.1091 | | | 0.3274 | |
| Cognition = Dementia | 0.632 | 0.189 – 1.074 | 0.0055 | | | 0.0164 | |
| APOE≥1 ε4 allele(s) | 0.204 | -0.022 – 0.43 | 0.0771 | | | 0.2314 | |
| History of Diabetes = Yes | 0.031 | -0.208 – 0.271 | 0.7955 | | | 1.0000 | |
| Race = Black:Aβ PET SUVR | 0.462 | -0.469 – 1.393 | 0.3284 | | | 0.9852 | |
| **Plasma p-tau217** | **β** | **95%CI** | **p** | | | **Adj p** | |
| Race = Black | -0.211 | -1.235 – 0.812 | 0.6838 | | | 1.0000 | |
| Aβ PET SUVR | 2.103 | 1.354 – 2.853 | <0.0001 | | | <0.0001 | |
| Age at Plasma (years) | 0.036 | 0.016 – 0.055 | 0.0004 | | | 0.0012 | |
| Plasma to AβPET | 0.278 | 0.119 – 0.437 | 0.0007 | | | 0.0022 | |
| ADI = Medium Deprivation | -0.192 | -0.425 – 0.041 | 0.1051 | | | 0.3153 | |
| ADI = High Deprivation | -0.039 | -0.351 – 0.272 | 0.8023 | | | 1.0000 | |
| Cognition = MCI | 0.328 | -0.008 – 0.665 | 0.0558 | | | 0.1673 | |
| Cognition = Dementia | 0.659 | 0.223 – 1.094 | 0.0033 | | | 0.0099 | |
| APOE≥1 ε4 allele(s) | 0.097 | -0.125 – 0.319 | 0.3892 | | | 1.0000 | |
| History of Diabetes = Yes | 0.063 | -0.173 – 0.299 | 0.5965 | | | 1.0000 | |
| Race = Black:Aβ PET SUVR | 0.203 | -0.713 – 1.119 | 0.6623 | | | 1.0000 | |
| **Plasma Aβ42/40** | **β** | **95%CI** | | **p** | **Adj p** | |  |
| Race = Black | 0.257 | -0.006 – 0.519 | | 0.0551 | 0.1654 | |  |
| Aβ PET SUVR | -0.178 | -0.37 – 0.014 | | 0.0693 | 0.2080 | |  |
| Age at Plasma (years) | -0.006 | -0.011 – -0.001 | | 0.0136 | 0.0409 | |  |
| Plasma to AβPET | 0.046 | 0.005 – 0.086 | | 0.0283 | 0.0850 | |  |
| ADI = Medium Deprivation | -0.043 | -0.103 – 0.016 | | 0.1545 | 0.4635 | |  |
| ADI = High Deprivation | -0.021 | -0.101 – 0.058 | | 0.5983 | 1.0000 | |  |
| Cognition = MCI | 0.037 | -0.049 – 0.123 | | 0.3994 | 1.0000 | |  |
| Cognition = Dementia | 0.028 | -0.084 – 0.139 | | 0.6265 | 1.0000 | |  |
| APOE≥1 ε4 allele(s) | -0.093 | -0.15 – -0.036 | | 0.0016 | 0.0047 | |  |
| History of Diabetes = Yes | -0.114 | -0.174 – -0.054 | | 0.0003 | 0.0008 | |  |
| Race = Black:Aβ PET SUVR | -0.143 | -0.378 – 0.092 | | 0.2316 | 0.6947 | |  |

##### **eTable 5. Interaction of Race by amyloid PET SUVR in Matched UPenn sample**. Linear models tested if the association of plasma biomarkers (log-transformed, dependent variable) with amyloid PET SUVR differed by self-identified race (White, Black/African American) (Interaction term: Race X amyloid PET SUVR). Covariates included age, area deprivation index (ADI), cognition (normal, impaired), and APOE ε4. Nominal and Bonferroni adjusted (Adj) *p*-values are reported.

## 5.4 Validation in ADNI

### 5.4.1 Classification of pBlack and pWhite


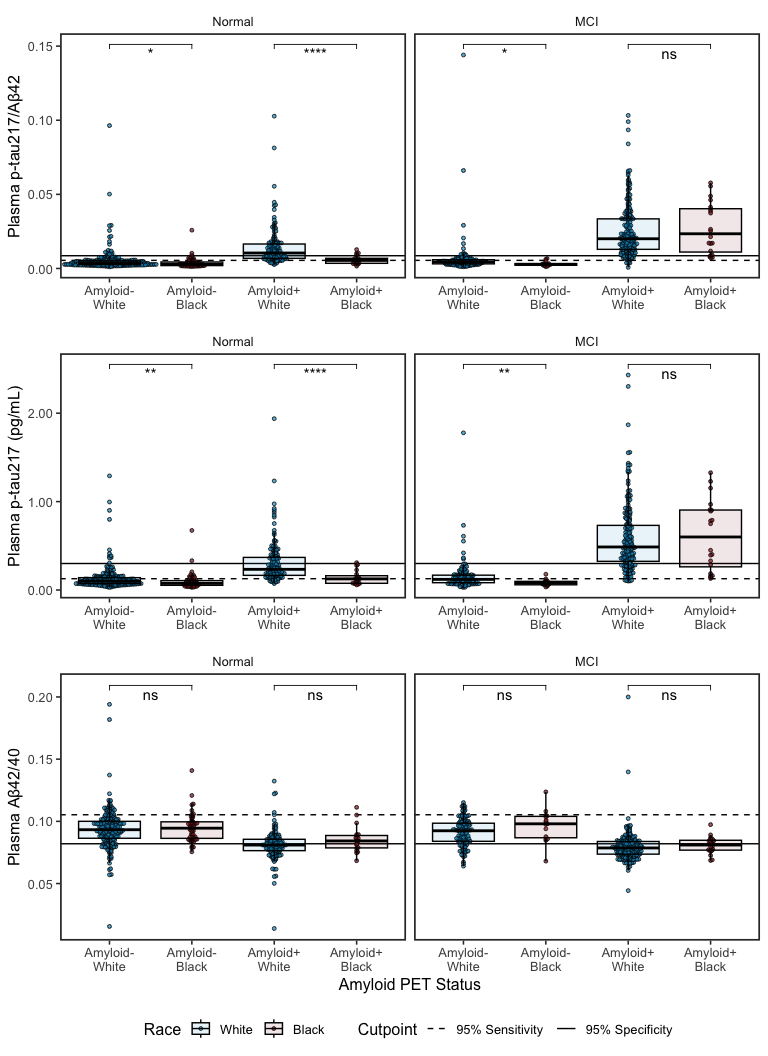


##### **eFigure 4. Plasma biomarkers by race (White, Black), Amyloid status (Amyloid+, Amyloid-), and cognitive status (normal, impaired) in all ADNI participants** (n=846). Boxplots show median, interquartile range (IQR), and outliers for each plasma biomarker. Color indicates self-identified White (blue) and Black/African American (red) participants. Left panels are cognitively normal/unimpaired individuals; right panels are individuals with cognitive impairment (*e.g.*, mild cognitive impairment, amnestic multidomain dementia, non-amnestic multidomain dementia). Solid horizontal line indicates 0.95 specificity threshold; broken horizonal lines indicate 0.95 sensitivity threshold (Table 1). Asterisks represent Bonferroni adjusted *p*-values from Wilcoxon pairwise comparisons (** Bonferroni-*p*<0.01, **** Bonferroni-*p*<0.001, **** Bonferroni-*p*<0.0001).

Excluded one Aβ42/Aβ40 outlier (0.66 in White Amyloid+ Normal individual) from figures for improved visualization, but was not excluded from statistical analyses.

| **A. Matched Performance** | Biomarker | Proportion Intermediate | Accuracy | 95% CI | Sens | Spec | PPV | NPV |
| --- | --- | --- | --- | --- | --- | --- | --- | --- |
| UPenn; White | p-tau217/Aβ42 | 0.12 | 0.91 | 0.82 – 0.97 | 0.85 | 0.94 | 0.85 | 0.94 |
| UPenn; Black | p-tau217/Aβ42 | 0.09 | 0.96 | 0.88 – 0.99 | 0.95 | 0.96 | 0.90 | 0.98 |
| ADNI; White | p-tau217/Aβ42 | 0.13 | 0.90 | 0.82 – 0.95 | 0.89 | 0.91 | 0.80 | 0.95 |
| ADNI; Black | p-tau217/Aβ42 | 0.16 | 0.88 | 0.79 – 0.94 | **0.72** | 0.95 | 0.88 | 0.88 |
| UPenn; White | p-tau217 | 0.25 | 0.92 | 0.82 – 0.97 | **0.79** | 0.96 | 0.85 | 0.94 |
| UPenn; Black | p-tau217 | 0.19 | 0.94 | 0.85 – 0.98 | 0.93 | 0.94 | 0.82 | 0.98 |
| ADNI; White | p-tau217 | 0.26 | 0.91 | 0.82 – 0.96 | 0.90 | 0.91 | **0.79** | 0.96 |
| ADNI; Black | p-tau217 | 0.23 | 0.86 | 0.77 – 0.93 | **0.62** | 0.96 | 0.88 | 0.86 |
| UPenn; White | Aβ42/Aβ40 | 0.58 | 0.88 | 0.73 – 0.97 | 1.00 | 0.85 | **0.67** | 1.00 |
| UPenn; Black | Aβ42/Aβ40 | 0.38 | 0.90 | 0.78 – 0.97 | **0.75** | 0.95 | 0.82 | 0.92 |
| ADNI; White | Aβ42/Aβ40 | 0.58 | **0.73** | 0.57 – 0.85 | 0.96 | **0.48** | **0.67** | 0.91 |
| ADNI; Black | Aβ42/Aβ40 | 0.69 | **0.76** | 0.58 – 0.89 | 0.94 | **0.56** | **0.70** | 0.90 |


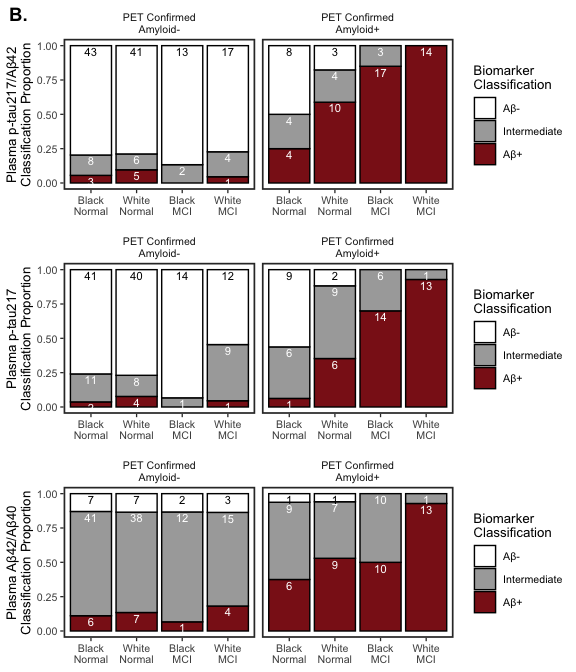


##### **eFigure 5: Application of biomarker thresholds in matched UPenn** (n=160; 80 pBlack, 80 pWhite) **and matched ADNI** (n=210; 105 pBlack, 105 pWhite). (**A.**) Derived from UPenn training set, thresholds determined “Aβ+”, “Aβ-”, and “intermediate” classifications. Thresholds were applied in matched UPenn and ADNI datasets. Performance metrics (Accuracy, Accuracy 95% confidence interval [95%CI], Sensitivity [Sens], Specificity [Spec], Positive Predictive Value [PPV], Negative Predictive Value [NPV]) are based on subjects classified as Aβ+ or Aβ-; performance values<0.80 are colored in red. Intermediate cases were excluded from performance calculations (Accuracy, 95%CI, Sens, Spec, PPV, NPV). (**B.**) Classification performance of biomarker thresholds by race in matched ADNI. Barplots show proportion of biomarker classifications as Amyloid- (white), Intermediate (grey), or Amyloid+ (red), by race (White, Black) and cognition (Normal, MCI). Left panels are Amyloid-; right panels are Amyloid+. Count for each classification are labeled in bars.”)

### 5.4.2 Group Differences in Matched ADNI Sample

| Matched ADNI Sample | White | Black | p | Missing |
| --- | --- | --- | --- | --- |
| n | 105 | 105 |  |  |
| Pet Status = Amyloid+ (%) | 31 (29.5%) | 36 (34.3%) | 0.554 | -- |
| Aβ PET Centiloid | 7.0 [-2.0, 36.0] | 9.0 [-1.0, 41.0] | 0.268 | -- |
| Cognitive Status = MCI (%) | 36 (34.3%) | 35 (33.3%) | 1.000 | -- |
| Age at Plasma (years) | 69.0 [65.0, 77.0] | 70.0 [65.0, 75.0] | 0.928 | -- |
| Body Mass Index (BMI) | 27.4 [24.0, 29.8] | 29.5 [25.5, 33.9] | 0.001 | 24 |
| Creatinine | 0.8 [0.7, 1.0] | 0.9 [0.7, 1.0] | 0.274 | 29 |
| Sex = Male (%) | 31 (29.5%) | 28 (26.7%) | 0.759 | -- |
| Ethnicity = Hispanic or Latino (%) | 7 (6.7%) | 1 (1.0%) | 0.074 | 1 |
| Education | 16.0 [14.0, 18.0] | 16.0 [14.0, 18.0] | 0.843 | -- |
| APOE = ≥1 ε4 allele(s) (%) | 31 (31.3%) | 40 (42.1%) | 0.158 | 16 |
| Area Deprivation Index (ADI) (%) |  |  | 0.026 | 106 |
| Low Deprivation | 21 (50.0%) | 24 (38.7%) |  |  |
| Medium Deprivation | 17 (40.5%) | 18 (29.0%) |  |  |
| High Deprivation | 4 (9.5%) | 20 (32.3%) |  |  |

##### **eTable 6: Demographic, pathological, and clinical characteristics of ADNI matched sample.** For continuous variables, median and interquartile range (IQR) are reported; Kruskal-Wallis tests performed group comparisons. For categorical variables, count (percentage [%]) are provided; chi-square tests performed frequency comparisons. *p*-values are reported for group comparisons.


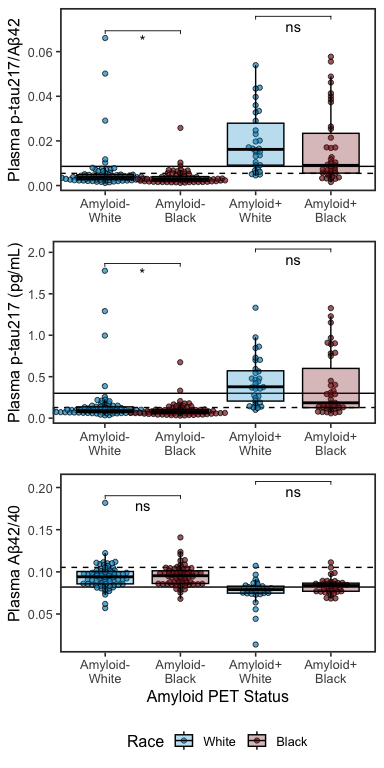


##### **eFigure 6. Plasma biomarkers in Amyloid+ and Amyloid-, by Race in Matched ADNI Sample** (n=210). Boxplots show median, interquartile range (IQR), and outliers for each plasma biomarker. Color indicates self-identified White (blue) and Black/African American (red) participants. Solid horizontal line indicates 0.95 specificity threshold; broken horizonal lines indicate 0.95 sensitivity threshold (Table 1). Asterisks represent Bonferroni *p*-values from Wilcoxon pairwise comparisons (* Bonferroni-*p*<0.05 or not significant [ns]).

| **Plasma p-tau217/Aβ42** | **β** | **95%CI** | **p** | | **Adj p** | | |  |
| --- | --- | --- | --- | --- | --- | --- | --- | --- |
| PET Status = Amyloid+ | 1.199 | 0.921 – 1.477 | <0.0001 | | <0.0001 | | |  |
| Race = Black | -0.181 | -0.402 – 0.041 | 0.1087 | | 0.3261 | | |  |
| Age at Plasma (years) | 0.011 | -0.004 – 0.026 | 0.1435 | | 0.4304 | | |  |
| Sex = Male | 0.194 | -0.083 – 0.47 | 0.1686 | | 0.5059 | | |  |
| Cognition = MCI | 0.393 | 0.165 – 0.621 | 0.0009 | | 0.0026 | | |  |
| APOE≥1 ε4 allele(s) | 0.125 | -0.128 – 0.377 | 0.3314 | | 0.9942 | | |  |
| Body Mass Index (BMI) | -0.015 | -0.031 – 0 | 0.0542 | | 0.1626 | | |  |
| log(Creatinine) | 0.371 | -0.14 – 0.882 | 0.1531 | | 0.4592 | | |  |
| **Plasma p-tau217** | **β** | **95%CI** | | **p** | | **Adj p** | | |
| PET Status = Amyloid+ | 1.107 | 0.839 – 1.374 | | <0.0001 | | <0.0001 | | |
| Race = Black | -0.238 | -0.451 – -0.025 | | 0.0288 | | 0.0864 | | |
| Age at Plasma (years) | 0.015 | 0.001 – 0.03 | | 0.0376 | | 0.1128 | | |
| Sex = Male | 0.057 | -0.21 – 0.323 | | 0.6753 | | 1.0000 | | |
| Cognition = MCI | 0.379 | 0.159 – 0.599 | | 0.0009 | | 0.0026 | | |
| APOE≥1 ε4 allele(s) | 0.031 | -0.212 – 0.275 | | 0.7985 | | 1.0000 | | |
| Body Mass Index (BMI) | -0.012 | -0.027 – 0.003 | | 0.1133 | | 0.3400 | | |
| log(Creatinine) | 0.748 | 0.256 – 1.24 | | 0.0031 | | 0.0094 | | |
| **Plasma Aβ42/Aβ40** | **β** | **95%CI** | | **p** | | **Adj p** |  |  |
| PET Status = Amyloid+ | -0.102 | -0.147 – -0.057 | | <0.0001 | | 0.0001 |  |  |
| Race = Black | 0.016 | -0.02 – 0.052 | | 0.3914 | | 1.0000 |  |  |
| Age at Plasma (years) | -0.001 | -0.003 – 0.001 | | 0.4420 | | 1.0000 |  |  |
| Sex = Male | -0.027 | -0.072 – 0.018 | | 0.2409 | | 0.7227 |  |  |
| Cognition = MCI | -0.015 | -0.053 – 0.022 | | 0.4117 | | 1.0000 |  |  |
| APOE≥1 ε4 allele(s) | -0.055 | -0.096 – -0.014 | | 0.0089 | | 0.0267 |  |  |
| Body Mass Index (BMI) | 0.000 | -0.003 – 0.002 | | 0.7092 | | 1.0000 |  |  |
| log(Creatinine) | 0.065 | -0.018 – 0.148 | | 0.1246 | | 0.3737 |  |  |
| **Plasma Aβ42** | **β** | **95%CI** | | **p** | | **Adj p** | | |
| PET Status = Amyloid+ | -0.092 | -0.161 – -0.023 | | 0.0089 | | 0.0268 | | |
| Race = Black | -0.057 | -0.112 – -0.003 | | 0.0404 | | 0.1211 | | |
| Age at Plasma (years) | 0.004 | 0 – 0.008 | | 0.0280 | | 0.0839 | | |
| Sex = Male | -0.137 | -0.205 – -0.069 | | 0.0001 | | 0.0004 | | |
| Cognition = MCI | -0.014 | -0.071 – 0.042 | | 0.6199 | | 1.0000 | | |
| APOE≥1 ε4 allele(s) | -0.093 | -0.156 – -0.031 | | 0.0038 | | 0.0115 | | |
| Body Mass Index (BMI) | 0.003 | -0.001 – 0.007 | | 0.1018 | | 0.3055 | | |
| log(Creatinine) | 0.377 | 0.25 – 0.503 | | <0.0001 | | <0.0001 | | |
| **Plasma Aβ40** | **β** | **95%CI** | | **p** | | **Adj p** | | |
| PET Status = Amyloid+ | 0.009 | -0.05 – 0.069 | | 0.7533 | | 1.0000 | | |
| Race = Black | -0.073 | -0.12 – -0.026 | | 0.0028 | | 0.0084 | | |
| Age at Plasma (years) | 0.005 | 0.002 – 0.008 | | 0.0020 | | 0.0059 | | |
| Sex = Male | -0.110 | -0.169 – -0.051 | | 0.0003 | | 0.0010 | | |
| Cognition = MCI | 0.001 | -0.048 – 0.05 | | 0.9598 | | 1.0000 | | |
| APOE≥1 ε4 allele(s) | -0.038 | -0.092 – 0.016 | | 0.1677 | | 0.5032 | | |
| Body Mass Index (BMI) | 0.004 | 0 – 0.007 | | 0.0304 | | 0.0911 | | |
| log(Creatinine) | 0.312 | 0.203 – 0.421 | | <0.0001 | | <0.0001 | | |

##### **eTable 7: Linear Models in Matched ADNI sample**. Linear models tested plasma biomarkers (log-transformed) as a function of Amyloid status (Amyloid-, Amyloid+) and self-identified race (White, Black), including age, sex, area deprivation index (ADI), cognition (normal, impaired), and APOE ε4 as covariates. Nominal and Bonferroni adjusted (Adj) *p*-values are reported.

### 5.4.3 Interaction of race by amyloid severity (measured by PET Centiloids)


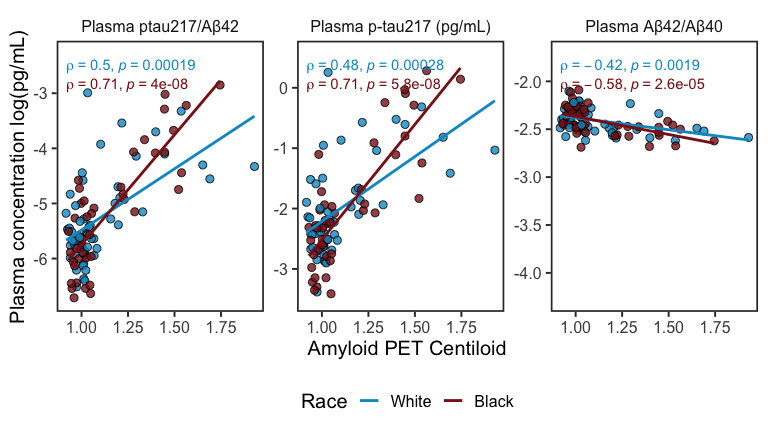


##### **eFigure 7. Associations of plasma biomarkers with amyloid severity by self-identified race in Matched ADNI sample** (n=210). Scatterplot of plasma biomarkers association with amyloid PET SUVR. Least squares regression lines are plotted. Color indicates self-identified White (blue) and Black/African American (red) participants. Spearman’s correlations with nominal *p*-values are reported.

| **Plasma ptau217/Aβ42** | **β** | **95%CI** | **p** | | **Adj p** | |
| --- | --- | --- | --- | --- | --- | --- |
| Race = Black | -0.253 | -0.487 – -0.019 | 0.0341 | | 0.1024 | |
| Aβ PET Centiloid | 0.019 | 0.014 – 0.025 | <0.0001 | | <0.0001 | |
| Age at Plasma (years) | 0.008 | -0.006 – 0.021 | 0.2761 | | 0.8283 | |
| Sex = Male | 0.176 | -0.08 – 0.431 | 0.1764 | | 0.5293 | |
| Cognition = MCI | 0.270 | 0.054 – 0.486 | 0.0148 | | 0.0445 | |
| APOE≥1 ε4 allele(s) | 0.127 | -0.098 – 0.353 | 0.2649 | | 0.7947 | |
| Body Mass Index (BMI) | -0.009 | -0.023 – 0.006 | 0.2343 | | 0.7028 | |
| log(Creatinine) | 0.393 | -0.075 – 0.86 | 0.0988 | | 0.2964 | |
| Aβ PET Centiloids:Race = Black | 0.000 | -0.007 – 0.007 | 0.9923 | | 1.0000 | |
| **Plasma p-tau217** | **β** | **95%CI** | **p** | | **Adj p** | |
| Race = Black | -0.306 | -0.528 – -0.083 | 0.0075 | | 0.0225 | |
| Aβ PET Centiloid | 0.018 | 0.013 – 0.023 | <0.0001 | | <0.0001 | |
| Age at Plasma (years) | 0.012 | -0.001 – 0.025 | 0.0785 | | 0.2354 | |
| Sex = Male | 0.042 | -0.201 – 0.285 | 0.7351 | | 1.0000 | |
| Cognition = MCI | 0.258 | 0.052 – 0.463 | 0.0145 | | 0.0435 | |
| APOE≥1 ε4 allele(s) | 0.024 | -0.191 – 0.238 | 0.8282 | | 1.0000 | |
| Body Mass Index (BMI) | -0.006 | -0.02 – 0.008 | 0.4037 | | 1.0000 | |
| log(Creatinine) | 0.769 | 0.324 – 1.214 | 0.0008 | | 0.0025 | |
| Aβ PET Centiloids:Race = Black | 0.000 | -0.006 – 0.006 | 0.9876 | | 1.0000 | |
| **Plasma Aβ42/40** | **β** | **95%CI** | **p** | **Adj p** | |  |
| Race = Black | 0.015 | -0.026 – 0.056 | 0.4750 | 1.0000 | |  |
| Aβ PET Centiloid | -0.002 | -0.003 – -0.001 | 0.0002 | 0.0006 | |  |
| Age at Plasma (years) | -0.001 | -0.003 – 0.002 | 0.5556 | 1.0000 | |  |
| Sex = Male | -0.027 | -0.072 – 0.018 | 0.2300 | 0.6901 | |  |
| Cognition = MCI | -0.008 | -0.046 – 0.03 | 0.6606 | 1.0000 | |  |
| APOE≥1 ε4 allele(s) | -0.058 | -0.097 – -0.018 | 0.0047 | 0.0140 | |  |
| Body Mass Index (BMI) | -0.001 | -0.003 – 0.002 | 0.4818 | 1.0000 | |  |
| log(Creatinine) | 0.065 | -0.017 – 0.147 | 0.1188 | 0.3563 | |  |
| Aβ PET Centiloids:Race = Black | 0.000 | -0.001 – 0.002 | 0.5366 | 1.0000 | |  |

##### **eTable 8. Interaction of Race by amyloid PET SUVR in Matched ADNI sample**. Linear models tested if the association of plasma biomarkers (log-transformed, dependent variable) with amyloid PET SUVR differed by self-identified race (White, Black/African American) (Interaction term: Race X amyloid PET SUVR). Covariates included age, area deprivation index (ADI), cognition (normal, impaired), and APOE ε4. Nominal and Bonferroni adjusted (Adj) *p*-values are reported.

### 5.4.4 Linear Combination of p-tau_217_ + Aβ_42_/Aβ_40_

Logistic regression tested the linear combination of log(p-tau_217_) + log(Aβ_42_/Aβ_40_) to Amyoid+ PET Status.

|  | AUC | AUC 95% CI | Method | Threshold | Sensitivity | Specificity | Accuracy |
| --- | --- | --- | --- | --- | --- | --- | --- |
| Plasma ptau217/Aβ42 | 0.95 | 0.91 – 0.97 | ≥0.95 sens | 0.0055 | 0.95 | 0.87 | 0.90 |
|  |  |  | ≥0.95 spec | 0.0086 | 0.84 | 0.95 | 0.91 |
| p-tau217 + Aβ42/Aβ40 | 0.94 | 0.91 – 0.97 | ≥0.95 sens | 0.2827 | 0.95 | 0.84 | 0.89 |
|  |  |  | ≥0.95 spec | 0.7255 | 0.71 | 0.95 | 0.85 |
| Plasma p-tau217 (pg/mL) | 0.93 | 0.89 – 0.96 | ≥0.95 sens | 0.1280 | 0.95 | 0.77 | 0.85 |
|  |  |  | ≥0.95 spec | 0.3000 | 0.67 | 0.95 | 0.83 |
| Plasma Aβ42/Aβ40 | 0.85 | 0.80 – 0.89 | ≥0.95 sens | 0.1053 | 0.95 | 0.44 | 0.65 |
|  |  |  | ≥0.95 spec | 0.0820 | 0.34 | 0.95 | 0.70 |

##### **eTable 9. Receiver Operating Characteristic (ROC) analyses in all UPenn to discriminate Amyloid+ from Amyloid-** (n=289). Bootstrapping with 2000 iterations computed ROC metrics. Area under the curve (AUC) and 95% confidence interval (95%CI) for each biomarker are reported. Maximum Youden’s index determined optimal threshold; sensitivity, specificity, and accuracy at thresholds are reported.

|  | Biomarker | Proportion Intermediate | Accuracy | 95% CI | Sens | Spec | PPV | NPV |
| --- | --- | --- | --- | --- | --- | --- | --- | --- |
| UPenn; All | p-tau217/Aβ42 | 0.09 | 0.95 | 0.91 – 0.97 | 0.94 | 0.95 | 0.93 | 0.96 |
| UPenn; White | p-tau217/Aβ42 | 0.10 | 0.94 | 0.90 – 0.97 | 0.94 | 0.94 | 0.93 | 0.95 |
| UPenn; Black | p-tau217/Aβ42 | 0.09 | 0.96 | 0.88 – 0.99 | 0.95 | 0.96 | 0.90 | 0.98 |
| ADNI; All | p-tau217/Aβ42 | 0.16 | 0.91 | 0.88 – 0.93 | 0.92 | 0.90 | 0.88 | 0.93 |
| ADNI; White | p-tau217/Aβ42 | 0.16 | 0.91 | 0.89 – 0.93 | 0.94 | 0.89 | 0.88 | 0.94 |
| ADNI; Black | p-tau217/Aβ42 | 0.16 | 0.88 | 0.79 – 0.94 | **0.72** | 0.95 | 0.88 | 0.88 |
| UPenn; All | p-tau217 + Aβ42/Aβ40 | 0.17 | 0.94 | 0.90 – 0.96 | 0.92 | 0.95 | 0.91 | 0.95 |
| UPenn; White | p-tau217 + Aβ42/Aβ40 | 0.17 | 0.92 | 0.87 – 0.96 | 0.92 | 0.93 | 0.91 | 0.94 |
| UPenn; Black | p-tau217 + Aβ42/Aβ40 | 0.15 | 0.97 | 0.90 – 1.00 | 0.94 | 0.98 | 0.94 | 0.98 |
| ADNI; All | p-tau217 + Aβ42/Aβ40 | 0.24 | 0.92 | 0.89 – 0.94 | 0.96 | 0.89 | 0.88 | 0.96 |
| ADNI; White | p-tau217 + Aβ42/Aβ40 | 0.25 | 0.92 | 0.89 – 0.94 | 0.97 | 0.87 | 0.87 | 0.97 |
| ADNI; Black | p-tau217 + Aβ42/Aβ40 | 0.23 | 0.91 | 0.83 – 0.96 | **0.75** | 0.98 | 0.95 | 0.90 |

##### **eTable 10: Application of linear combination thresholds (eTable 9) in ADNI test samples.** Plasma p-tau_217_/Aβ_42_ ratio and linear combination of log(p-tau_217_) + log(Aβ_42_/Aβ_40_) performance in ADNI samples (n=846). Performance metrics (Accuracy, Accuracy 95% confidence interval [95%CI], Sensitivity [Sens], Specificity [Spec], Positive Predictive Value [PPV], Negative Predictive Value [NPV]) are based on subjects classified as Aβ+ or Aβ-; performance values<0.80 are colored in red.”)


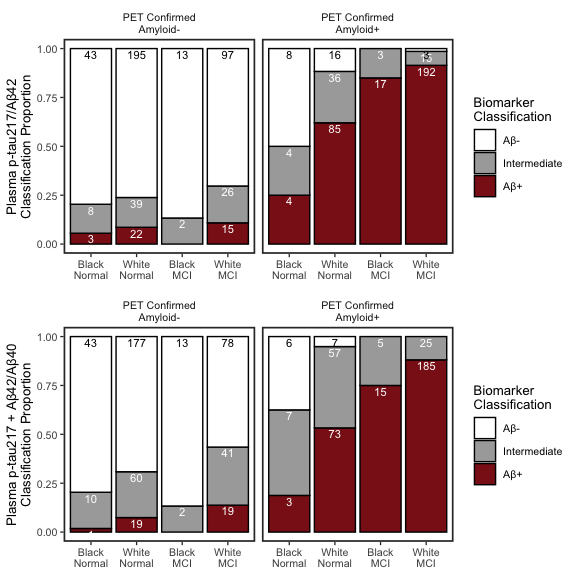


##### **eFigure 8: Application of ratio vs. linear combination thresholds in ADNI test set** (n=846). Classification performance of biomarker thresholds by race in ADNI for the p-tau_217_/Aβ_42_ ratio and linear combination of log(p-tau_217_) + log(Aβ_42_/Aβ_40_). Barplots show propotion of biomarker classifications as Amyloid- (white), Intermediate (grey), or Amyloid+ (red), by race (White, Black) and cognition (Normal, MCI). Left panels are Amyloid-; right panels are Amyloid+. Count for each classification are labeled in bars.”

### 5.4.5 One cutpoint strategy

| **One cutpoint in UPenn** | AUC | AUC 95% CI | Threshold | Threshold 95% CI | Sensitivity | Specificity | Accuracy |
| --- | --- | --- | --- | --- | --- | --- | --- |
| Plasma ptau217/Aβ42 | 0.95 | 0.91 – 0.97 | 0.0066 | 0.0057 – 0.0076 | 0.92 | 0.91 | 0.92 |
| Plasma p-tau217 (pg/mL) | 0.93 | 0.89 – 0.96 | 0.1689 | 0.1478 – 0.1907 | 0.90 | 0.88 | 0.89 |
| Plasma Aβ42/Aβ40 | 0.85 | 0.80 – 0.89 | 0.0943 | 0.0926 – 0.0965 | 0.80 | 0.79 | 0.80 |

##### **eTable 11. Receiver Operating Characteristic (ROC) analyses in all UPenn to discriminate Amyloid+ from Amyloid-** (n=289). Bootstrapping with 2000 iterations computed ROC metrics. Area under the curve (AUC) and 95% confidence interval (95%CI) for each biomarker are reported. Maximum Youden’s index determined optimal threshold; sensitivity, specificity, and accuracy at thresholds are reported.

|  | Biomarker | Accuracy | 95% CI | Sens | Spec | PPV | NPV |
| --- | --- | --- | --- | --- | --- | --- | --- |
| ADNI; All | p-tau217/Aβ42 | 0.85 | 0.83 – 0.88 | 0.88 | 0.84 | 0.82 | 0.89 |
| ADNI; White | p-tau217/Aβ42 | 0.86 | 0.83 – 0.88 | 0.90 | 0.83 | 0.82 | 0.90 |
| ADNI; Black | p-tau217/Aβ42 | 0.82 | 0.73 – 0.89 | **0.69** | 0.88 | **0.76** | 0.85 |
| ADNI; All | p-tau217 | 0.83 | 0.81 – 0.86 | 0.85 | 0.82 | 0.80 | 0.87 |
| ADNI; White | p-tau217 | 0.84 | 0.81 – 0.86 | 0.87 | 0.80 | 0.80 | 0.88 |
| ADNI; Black | p-tau217 | 0.81 | 0.72 – 0.88 | **0.58** | 0.93 | 0.81 | 0.81 |
| ADNI; All | Aβ42/Aβ40 | **0.67** | 0.64 – 0.71 | 0.94 | **0.46** | **0.59** | 0.90 |
| ADNI; White | Aβ42/Aβ40 | **0.68** | 0.64 – 0.71 | 0.95 | **0.44** | **0.60** | 0.90 |
| ADNI; Black | Aβ42/Aβ40 | **0.65** | 0.55 – 0.74 | 0.89 | **0.52** | **0.49** | 0.90 |

##### **eTable 12: Application of single biomarker threshold (eTable 9) in ADNI test samples.** Biomarker threshold performance in ADNI samples (n=846). Single thresholds based on Youden’s index are applied from UPenn sample (eTable 9). Performance metrics (Accuracy, Accuracy 95% confidence interval [95%CI], Sensitivity [Sens], Specificity [Spec], Positive Predictive Value [PPV], Negative Predictive Value [NPV]) are based on subjects classified as Aβ+ or Aβ-; performance values<0.80 are colored in red.”)

### 5.4.6 Dataset comparison

| **A. pBlack** | UPenn | ADNI | p | Missing |
| --- | --- | --- | --- | --- |
| n | 80 | 105 |  |  |
| AStatus = Aβ+ (%) | 21 (26.2%) | 36 (34.3%) | 0.312 | -- |
| Age at Plasma (years) | 72.000 [69.000, 76.000] | 70.000 [65.000, 75.000] | 0.005 | -- |
| Body Mass Index (BMI) | 29.742 [26.193, 34.605] | 29.528 [25.473, 33.865] | 0.666 | 39 |
| Sex = Male (%) | 9 (11.2%) | 28 (26.7%) | 0.016 | -- |
| Education | 16.000 [12.750, 18.000] | 16.000 [14.000, 18.000] | 0.390 | -- |
| APOE = ≥1 ε4 allele(s) (%) | 34 (43.0%) | 40 (42.1%) | 1.000 | 17 |
| Area Deprivation Index (ADI) (%) |  |  | 0.261 | 72 |
| Low Deprivation | 20 (26.0%) | 24 (38.7%) |  |  |
| Medium Deprivation | 29 (37.7%) | 18 (29.0%) |  |  |
| High Deprivation | 28 (36.4%) | 20 (32.3%) |  |  |
| Plasma p-tau217 (pg/mL) | 0.104 [0.066, 0.234] | 0.099 [0.063, 0.166] | 0.556 | -- |
| Plasma Aβ42/Aβ40 | 0.103 [0.092, 0.116] | 0.088 [0.084, 0.098] | <0.001 | -- |
| Plasma ptau217/Aβ42 | 0.003 [0.002, 0.009] | 0.004 [0.002, 0.007] | 0.276 | -- |
| **B. pWhite** | UPenn | ADNI | p | Missing |
| n | 209 | 741 |  |  |
| AStatus = Aβ+ (%) | 99 (47.4%) | 347 (46.8%) | 0.952 | -- |
| Cognitive Status (%) |  |  | <0.001 | -- |
| Normal | 113 (54.1%) | 393 (53.0%) |  |  |
| MCI | 71 (34.0%) | 348 (47.0%) |  |  |
| Dementia | 25 (12.0%) | 0 |  |  |
| Age at Plasma (years) | 73.000 [69.000, 78.000] | 74.000 [69.000, 79.000] | 0.072 | -- |
| Body Mass Index (BMI) | 25.683 [22.708, 29.022] | 26.363 [23.750, 29.639] | 0.087 | 36 |
| Sex = Male (%) | 101 (48.3%) | 397 (53.6%) | 0.206 | -- |
| Education | 18.000 [16.000, 18.000] | 16.000 [15.000, 18.000] | 0.050 | -- |
| APOE = ≥1 ε4 allele(s) (%) | 82 (39.2%) | 312 (43.5%) | 0.307 | 7 |
| Area Deprivation Index (ADI) (%) |  |  | <0.001 | 150 |
| Low Deprivation | 127 (66.5%) | 148 (55.8%) |  |  |
| Medium Deprivation | 58 (30.4%) | 79 (29.8%) |  |  |
| High Deprivation | 6 (3.1%) | 38 (14.3%) |  |  |
| Plasma p-tau217 (pg/mL) | 0.166 [0.079, 0.409] | 0.174 [0.102, 0.386] | 0.227 | -- |
| Plasma Aβ42/Aβ40 | 0.094 [0.085, 0.103] | 0.085 [0.078, 0.095] | <0.001 | -- |
| Plasma ptau217/Aβ42 | 0.006 [0.003, 0.015] | 0.007 [0.004, 0.016] | 0.027 | -- |

##### **eTable 13: Demographic and clinical characteristics of training (UPenn) vs. test (ADNI).** For continuous variables, median and interquartile range (IQR) are reported; Kruskal-Wallis tests performed group comparisons. For categorical variables, count (percentage [%]) are provided; chi-square tests performed frequency comparisons. *p*-values are reported for group comparisons.
